# Exploring Explanations of Subglacial Bedform Sizes Using Statistical Models

**DOI:** 10.1371/journal.pone.0159489

**Published:** 2016-07-26

**Authors:** John K. Hillier, Ioannis A. Kougioumtzoglou, Chris R. Stokes, Michael J. Smith, Chris D. Clark, Matteo S. Spagnolo

**Affiliations:** 1 Department of Geography, Loughborough University, Loughborough, United Kingdom; 2 Department of Civil Engineering and Engineering Mechanics, Columbia University, New York, New York, United States of America; 3 Department of Geography, Durham University, Durham, United Kingdom; 4 School of Geography, Earth and Environment, Kingston University, Kingston upon Thames, United Kingdom; 5 Department of Geography, University of Sheffield, Sheffield, United Kingdom; 6 School of Geosciences, University of Aberdeen, Aberdeen, United Kingdom; Louisiana State University, UNITED STATES

## Abstract

Sediments beneath modern ice sheets exert a key control on their flow, but are largely inaccessible except through geophysics or boreholes. In contrast, palaeo-ice sheet beds are accessible, and typically characterised by numerous bedforms. However, the interaction between bedforms and ice flow is poorly constrained and it is not clear how bedform sizes might reflect ice flow conditions. To better understand this link we present a first exploration of a variety of statistical models to explain the size distribution of some common subglacial bedforms (i.e., drumlins, ribbed moraine, MSGL). By considering a range of models, constructed to reflect key aspects of the physical processes, it is possible to infer that the size distributions are most effectively explained when the dynamics of ice-water-sediment interaction associated with bedform growth is fundamentally random. A ‘*stochastic instability*’ (SI) model, which integrates random bedform growth and shrinking through time with exponential growth, is preferred and is consistent with other observations of palaeo-bedforms and geophysical surveys of active ice sheets. Furthermore, we give a proof-of-concept demonstration that our statistical approach can bridge the gap between geomorphological observations and physical models, directly linking measurable size-frequency parameters to properties of ice sheet flow (e.g., ice velocity). Moreover, statistically developing existing models as proposed allows quantitative predictions to be made about sizes, making the models testable; a first illustration of this is given for a hypothesised repeat geophysical survey of bedforms under active ice. Thus, we further demonstrate the potential of size-frequency distributions of subglacial bedforms to assist the elucidation of subglacial processes and better constrain ice sheet models.

## 1. Introduction

Observations of palaeo-ice sheet beds show sediment that is commonly organized into subglacial bedforms (e.g., drumlins), whose shape or occurrence is thought to reflect ice flow conditions [1–3]. Concurrently, these bedforms are also thought to modulate ice flow characteristics, such as velocity (*v*) through their effect on subglacial hydrology, basal friction and roughness [4–7]. In short, there is likely an association between bedform morphology and the behaviour of the ice-sediment-water system that drives their formation.

Recently, geophysical observations from an Antarctic ice stream have revealed bed conditions [8–10] and bedforms that evolve, grow, and shrink on sub-decadal timescales [11–14]. However, these observations are logistically challenging and so limited to relatively few bedforms at one site [13,14]. In contrast, palaeo-bedforms are abundant (i.e., > 100,000s) and widespread, but it is more challenging to link them securely to processes at the ice sheet bed. Thus, our understanding of the processes occurring beneath contemporary ice sheets is incomplete, with some fundamental questions largely unanswered, e.g., how do bedforms grow, evolve their shape (e.g., elongate), regulate sediment flux, and interact with basal conditions such as 'sticky spots' (e.g., [15])?

**Fig 1:**
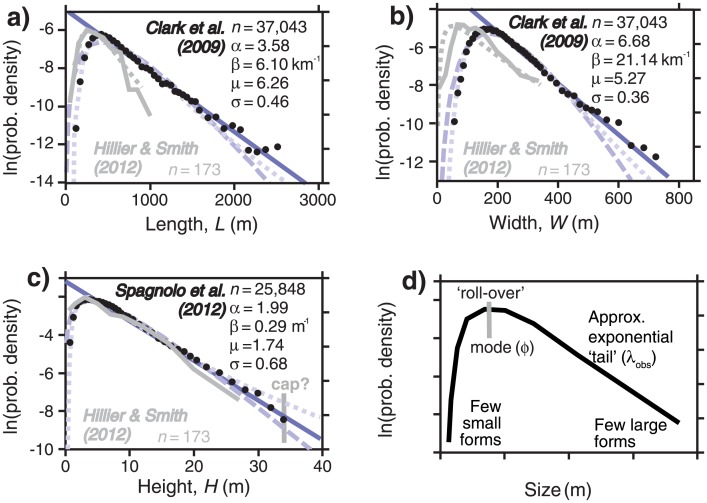
Size-frequency data and statistical distributions fitted to them. a) to c) Normalised histograms of observed drumlin attributes on semi-log plots (black dots), to which selected statistical distributions are fitted and plotted as probability density functions (pdfs); exponential distribution (solid blue line); gamma distribution (dashed line) (α_obs_, β_obs_) [19]; log-normal (dotted line) (μ_obs_, σ_obs_) [22]. Fits to obtain the distribution parameters, shown as Greek letters, are performed using estimators (e.g., maximum likelihood) as detailed in Appendix B. Data source and number of observed bedforms *n* are indicated on the plots; country-wide UK data (Fig 8 in [16] and Fig 5 in [31]) (black) and a well-studied sub-set (grey) of this [32] are used. d) The typical shape; there are few small bedforms, a modal peak above this forming a `roll-over’, and an approximately exponential tail of frequencies decreasing towards the largest sizes.

**Fig 2:**
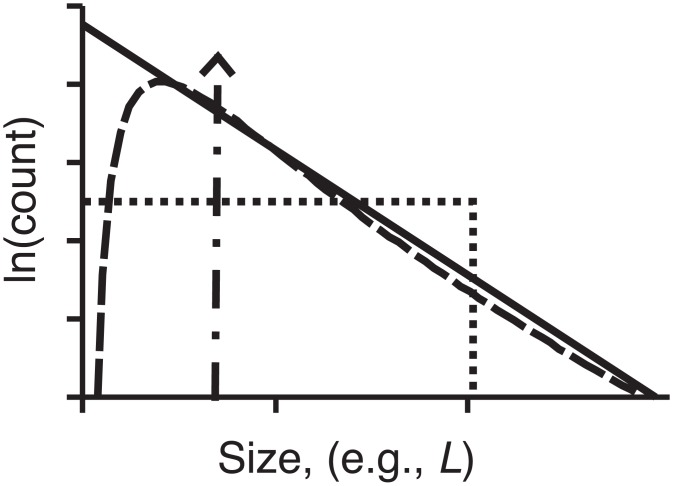
Illustrative size-frequency distributions from statistical growth models. Semi-log frequency plot illustrating a variety of size-frequency distributions of bedforms predicted by different types of statistical growth model. They are each governed by arguably plausible glaciological or statistical assumptions (see text for models): Dirac delta function (dot-dash line is Model 1, denoted M1); uniform distribution (dotted line e.g., M4); exponential (solid line e.g., M8); log-normal (dashed line e.g., M7). The power of this size-frequency data as a constraint is that only a sub-set of models produces distributions reasonably approximating observed data (e.g., Fig 1).

Size-frequency statistics of observed groups of bedforms thought to be genetically linked (Fig 1), known as ‘flow sets’ (e.g., [16]) or ‘fans’ [17], may provide an additional powerful constraint on such questions (e.g., [18,19]). However, these statistics are under-exploited, and factors such as the shape of the frequency distribution have been given only limited attention. Distribution shape has been neglected as a constraint because the current conceptual and physics-based models do not predict bedform size-frequency distributions. The potential to act as a constraint arises because not all conceptual or physics-based models (e.g., [20,21]) explaining bedform growth will replicate the observed sizes. Statistical models [19,22], however, have the potential to predict bedform sizes as a combined product of key aspects of the physical process: antecedent bedform-scale topography, growth rate (e.g., exponential), and the timing of growth. Fig 2 illustrates size distributions produced by a variety of statistical models, some of which are consistent with the shape of observed distributions and some are not.

Hillier et al. [19] first proposed a conceptual model to explain subglacial bedforms' size-distributions, in which ice-sediment-water interaction creating bedforms is fundamentally stochastic. Specifically, to explain an exponential tail to the size-distribution, this model suggests that bedform growth processes may be a convolution of randomness with simple rules about their rate of growth; analogous models of 'self-organized criticality' are used to explain power-law distributions [23,24]. The subglacial model draws upon ideas of probabilistic sediment transport [25] and an analogy to fluvial bedforms whose heavy-tailed size-distributions are thought to originate through growth in the presence of random fluctuations associated with turbulent flow [26–30]. As a concept this is consistent with the geophysical observations in Antarctica, but does not necessarily exclude either ice-till (e.g., [20]) or meltwater (e.g., [21]) bedform growth models. Fowler et al. [22] formalized a first statistical model of bedform sizes, investigating explanations for the particular case of a log-normal approximation to the observed size-distribution under the assumption of exponential growth without shrinking. This paper, to better understand how bedform sizes might reflect ice flow conditions, re-formulates and develops Fowler's statistical model and creates a new range of other models. This variety of models is a first exploration of the possibilities and allows, by putting each model in context, an assessment of its relative plausibility.

The paper begins by describing the size-frequency observations of bedforms (i.e., drumlins, ribbed moraine, MSGL), then outlines the terminology and defines a conceptual framework necessary for statistically modelling the evolution of sets of such subglacial bedforms. It then builds new statistical models, which are evaluated and discussed in light of observational evidence, internal consistency, and their implications for theories of bedform growth and the ice-water-sediment system under ice sheets. In addition, the models are shown to make distinctive predictions that could be tested should a geophysical survey under active ice (i.e., [13]) be repeated. Because growth in bedform height (*H*) underlies most physical modelling (e.g., [20,33,34]) the models are initially developed for height, but with implications for width (*W*) and length (*L*) also discussed.

## 2. Size Observations

Fig 1 illustrates typical size-frequency statistics of observed groups of subglacial bedforms. Distribution shapes are similar across bedform types (i.e., drumlins, MSGL, ribbed moraine), mappers and regions (e.g. UK, Canada, Sweden) [19]. Although a selection of statistical distributions could be fitted to bedform size data (e.g., [26]), subglacial bedform sizes have been found to be reasonably approximated as having a log-normal shape [22,35,36] or as being exponential above their mode [19]. Large compilations of bedforms (*n* > 10,000) (e.g., [16]) more precisely constrain their size distribution than smaller ones as uncertainty in sampling is reduced, but almost certainly represent the aggregation of a range of subglacial conditions. As such, the size distributions of large compilations may simply represent the statistical effects of aggregating samples rather than anything to do with ice flow. It is therefore important to note that the same distribution shape and spread of sizes is still apparent within flow-sets comprising 100–200 bedforms (Fig 1, grey lines) that likely represent something about glaciological conditions at a particular location in space and time.

The parameters listed in Fig 1 for the best-fitting gamma (α, β) and log-normal (μ, σ) distributions are obtained by method of moment and maximum likelihood estimators as described in Appendix B. Country-wide UK data in Fig 1 are, quite deliberately, values digitised from plots in the original papers [16,31]. This is done to demonstrate that the published archive of size-distributions can be usefully re-assessed in light of statistical models. Parameters calculated from digitized values typically differ little from those used to construct the original plots (e.g., <3% for μ and σ). Furthermore, the data of Hillier and Smith [32] show that parameter values are similar when calculated from either counts within size bins or from the individual underlying data (e.g., variations <7% for μ and σ). Importantly, patterns in relative values (e.g., σ_H_>σ_W_>σ_L_) are robustly unchanged for all parameters, and the differences between their values (e.g., for *H* vs. *W*) are always substantially larger than uncertainties caused by the method used to derive the parameter values (see S1 File).

Initially, the parameters are simply empirical descriptors of the shape of the size-frequency distributions; it is statistical models of bedform growth that potentially allow the parameters to be considered in terms of subglacial processes. A conceptual framework is now created, which outlines the elements necessary to formulate statistical models that might explain the observed size-frequency distributions.

## 3. Conceptual Framework

Firm and direct observational constraints on how glacial bedforms are formed have proved challenging to obtain. However, to formalise statistical models, a framework is needed. Geophysical surveys [11,13], sediment flux estimates [37], and geometric arguments [38] indicate that forms entirely composed of sediment could arise over ~10s-100s years, and certainly within one ice flow event (e.g., [39,40]). Thus, modelling can start by considering one flow episode. However, substantial elements of the processes at work remain unclear. How do bedforms initiate? Do initial sizes determine final ones? Is growth exponential with time, characteristic of linear instability? Is growth continuous or discrete, and monotonic or fluctuating, over time? Are bedforms in equilibrium with ice flow? It is not practical to model all views held on these questions, so these topics are introduced in order to highlight the choices made in constructing the statistical models.

### 3.1. Bedform initiation: growth and location

Entirely bedrock bedforms exist, and require an erosional mechanism (e.g., [41]). The majority, however, appear to be composed mainly or entirely of glacially-derived sediment (i.e., till) [42,43] requiring a mechanism for an origin from a till sheet (e.g., see [44]); this could involve erosion, deposition or redistribution or a combination of any of these processes (e.g., [45]). Subglacial bedforms might decrease in height from some set of progenitor forms (e.g., [46]). Alternatively, if sculpted from a relatively flat surface, they must (as a net effect over a period of time) increase their amplitude or ‘grow’ (e.g., [20]). This paper considers a sub-set of statistical models of bedform genesis in which bedforms undergo net growth, including models that incorporate periods were bedforms are stable or shrink. The mechanism of net growth may be till deformation (e.g., [47,48]) but, especially in light of studies into the size distribution of fluvial scours (e.g., [49]), the statistical models may also apply to conceptual models of the ice-sediment-water system governed by erosion or scour by meltwater (e.g., [21,50,51]).

**Fig 3:**
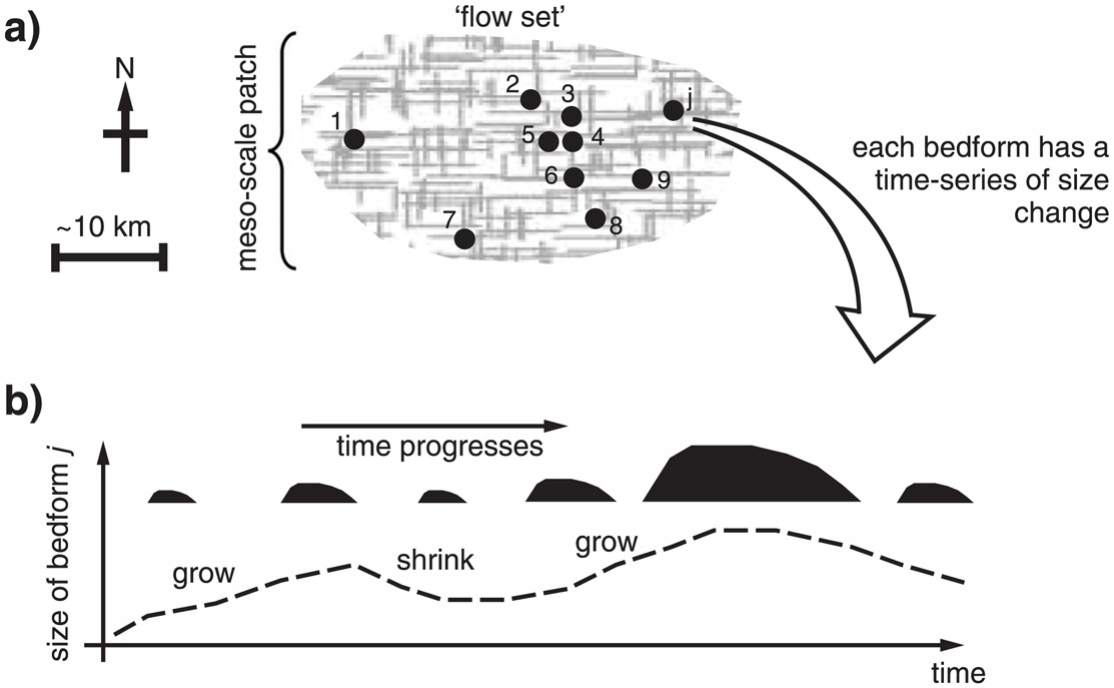
Conceptualisation of how flow-sets of bedforms grow. a) Cross-hatched area is a meso-scale flow-set (~10–100 km) or `patch’ of deformable or erodible subglacial material subjected to conditions conducive to a flow set of bedforms arising in locations illustrated by black dots. Within this, bedforms from 1 to *j*, where *j* is any integer, change in amplitude through erosion, deposition, or redistribution. b) A potential, illustrative, sequence of growth for one bedform (number *j*) through time (dashed line), accompanied by selected silhouettes representing vertical cross-sections; a shrinking rate of zero (i.e., stasis) is valid within the illustration.

It is known that bedforms occur more densely in some places than others, creating patchiness on a scale of 10-100s of km (e.g., [52,53]). ‘Patches’ defined in this way encompass numerous individual bedforms, which are typically 0.1–10 km in horizontal extent. Thus, meso-scale (~10-100s km) ‘*patches*’ are envisaged for the statistical models (Fig 3), which contain a statistically useful number (i.e., 1,2,3 … *j*) of bedforms linked to relatively local conditions (black dots) that grow in height (i.e., *H*). The premise of using patches as defined is consistent with the idea of spatio-temporally variable mosaics of stable and deforming bed conditions; this is based on observations of exposed till [54,55], but also consistent with geophysical studies that have revealed variable bed conditions [9,10]. Spatial variation in conditions is also postulated in bedform models that invoke meltwater [56].

### 3.2. Growth style: deterministic versus probabilistic

‘*Deterministic*’ growth is where proto-bedforms of a given size and shape always evolve similarly with time to a predictable final morphology; i.e., initial conditions lead uniquely to a final configuration. ‘*Probabilistic*’ growth is where random variability through time (i.e., dynamics) causes individual bedforms to evolve unpredictably or ‘*stochastically*’, but combine to produce predictable flow set statistics (e.g., [18,57]). In the non-turbulent conditions of ice flow, such variability is likely to arise from time-varying boundary conditions in the coupled ice-sediment-water system (e.g., water incursions, floods, basal stick-slip events) [58–61] or interactions between bedforms [62] perhaps by ice rheology inducing lateral stresses (e.g., [63,64]). Combining this with the observed range of time-scales on which ice flow fluctuates (i.e., days to decades) (e.g., [60,65–74]), and by analogy with established ideas in fluvial and aeolian environments (e.g., [25,28–30,57,62]), gives a picture of potentially pervasive randomness through time in subglacial sediment transport (i.e., flux) [19]. Either deterministic or probabilistic growth can be readily incorporated into statistical models.

### 3.3. Growth rate

Bedform growth predicted by physics-based models proceeds at a rate that has an expected characteristic mathematical form. If models relate till flux to the thickness of the till body and an unconnected ‘field’ variable, such as basal shear stress (*τ*), that can vary in space (e.g., [20,75,76]), growth of *H* is initially linear with time at a constant rate (*k*). In this regard *H* is governed by the ordinary differential equation (ODE)

$$\frac{\text{d}H}{\text{d}t}=k$$ (1)

in conjunction with the initial condition

$$H\left(t_{\text{i}}\right)=H_{i}.$$ (2)

Integrating Eq 1. analytically, considering the initial condition, and for final height denoting *H*(*t*_f_) = *H*_f_, yields Eq 3.

$$H_{\text{f}}=H_{i}+k\left(t_{\text{f}}-t_{\text{i}}\right)$$ (3)

If, on the other hand, models contain positive linear feedback between bedform and ‘field’ (Eq 4), this results in a physical instability in the sediment-ice system and growth is initially exponential with time (Eq 5) (e.g., [20,33]). Thus, the term ‘instability’ has been adopted to describe this class of sediment growth model. Note that the term instability is used in this way in this paper and not as strictly defined in the mathematical field of stability theory related to dynamics.

In this regard, where physical processes are thought to be approximated by linear feedback, *H* is governed by the ODE

$$\frac{\text{d}H}{\text{d}t}=kH$$ (4)

in conjunction with the initial condition of Eq 2. Similarly, as with Eq 1, integrating analytically yields

$$H_{\text{f}}=H_{\text{i}}e^{k\left(t_{\text{f}}-t_{\text{i}}\right)}$$ (5)

It is entirely plausible that growth does not continue according to either of these simple rate laws, perhaps because of ‘shock formation’ as *H* increases, which is when a subglacial bedform is dramatically altered after an ice-free cavity is generated on its lee side (e.g., [77,78]). The statistical models proposed below focus on the simple rate laws as it is not yet even well determined which of these might apply (cf. [79,80,81]). The models are, however, presented initially in terms of time spent growing so that they can be readily adapted for other rate laws if required in the future.

### 3.4. Continuous process versus discrete events

If bedform growth is viewed as a continuous property extending over a finite time period (e.g., [20,48,79]) then at any time, and for finite proportions of it, bedforms either grow or shrink. In contrast, and by analogy with other environments (e.g., [82,83]), the creation of each bedform may occur through discrete sediment flux '*events*', each of which might affect several proximal bedforms. However, if events affect only sub-areas of a patch and are randomly located, their impacts upon each bedform will appear as a series of independent trials through time [22], analogous to continuous variability. Thus, and particularly because analogies between the continuous and discrete mathematics exist (e.g., [84]), either a continuous or discrete modelling approach remains valid.

### 3.5. Transient versus equilibrium growth

The length of time over which a flow-set develops is not well constrained. It is therefore necessary to introduce into this framework the concept of ‘*transient*’ flow-set growth within a time window, between an initial time (*t*_i_) and a final time (*t*_f_). Pre-equilibrium or transient growth is where the statistics of a flow-set evolve over time, continue to evolve, and would have continued to evolve further if the conditions for growth had persisted. This contrasts to stable long-term ‘*equilibrium*’ behaviour in which the statistical characteristics of a flow set stabilise. Equilibrium is actively sought in fluvial experimentation (e.g., [26]) and has been implicitly invoked to infer ice properties; for example, assumed equilibrium is implicit when arguing that bedform elongation is related to ice velocity, rather than duration of flow (e.g., [3,85]). Bedforms that develop slowly with respect to changes in ice flow conditions at the flow-set scale (~10–100 km) will have pre-equilibrium transient statistics, whilst forms evolving much more rapidly than patch-scale flow changes could attain equilibrium. Which behaviour predominates amongst glacial bedforms is not yet known. Thus, statistical models containing both behaviours are permitted and explored here.

## 4. Methods

To better understand how bedform sizes might reflect formative flow conditions a new range of statistical models are developed, including one that extends the model of Fowler et al [22]. This variety of models allows, by putting each model in context, an assessment of its relative plausibility. The initial mode of discrimination is by the shape of the size-frequency distribution that each model creates (e.g., Fig 2) as compared to observations. Specifically, as also demonstrated in Fig 1, the data are reasonably approximated by log-normal [22,35,36] and gamma distributions, and by an exponential tail above the mode [19]. Models are therefore required to generate at least one of these to be considered as potentially plausible. Models are developed analytically so that the form of the size-frequency distributions they can produce is known explicitly.

## 5. Models

**Fig 4:**
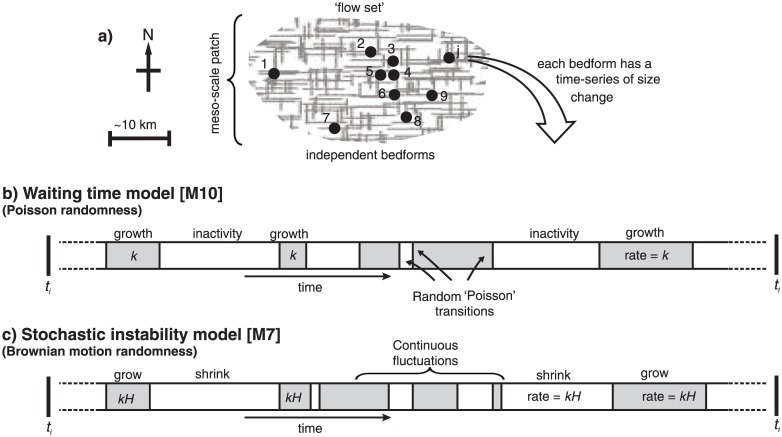
Framework for the statistical models. Cross-hatched area in a) is a meso-scale (~10s-100s km) ‘patch’ of deformable or erodible subglacial material subject to conditions conducive to a flow set of bedforms arising. b) and c) are barcode style strips for the waiting time (WT) [M10] and stochastic instability (SI) [M7] models. The strips represent the size evolution through time for one of the bedforms *j* in a). Specifically, the bands represent alternating `local’ (~0.1–1 km) conditions affecting *H*; grey is growth, and white is shrinking or inactivity. *k* and *kH* indicate growth rate (i.e., Eqs 1 and 4). Rapid fluctuations in c) are omitted for visual clarity, analogous to a time-series recorded at low temporal resolution.

The models developed here contain a number (i.e., 1,2,3 … *j*) of non-overlapping bedforms (Fig 4a, black dots) characterised as growing independently for a time period between *t*_i_ and *t*_f_ within 'meso-scale’ (~10-100s km) ‘*patches*’ when an appropriate flow regime prevails. Statistical independence between bedforms is assumed as in previous statistical modelling (i.e., [22]), where it is justified by randomness in the perturbing field (e.g., water influx) (see Section 3.4), although it may also be augmented by spatial randomness in rheological properties (e.g., viscosity). This is consistent with stochastic sediment flux in aeolian cellular-automata models that has yielded randomly sized, yet spatially patterned, barchan dunes [62,86]. Effective independence is also supported by analogy to extensive work in the fluvial environment where the growth of spatially ordered and self-organized bedforms is statistically described and modelled as stochastic and random [26,28,30,57,87]. We acknowledge that, with limited observational evidence, this set-up may not ultimately turn out to be correct, but it forms a useful basis to start an exploration with statistical models. Physically, activity within the patches is conceptualised as being based on multiple, rapid (i.e., sub-decadal) and random fluctuations in basal conditions that generate flow sets of bedforms.

Models are numbered, so that Model 4 is denoted [M4], for example. Each includes four elements, a growth rate 'law' based upon suggestions from physical models [20,33,75,76], rules about what initial sizes are and when growth begins, and a growth style that is deterministic or uses temporal randomness. Each aspect affects the output size distribution, and the characteristics of all models are summarised in Table 1. The simplest new models created, both mathematically and conceptually, are those that do not involve stochasticity in growth through time [M1-5]. Some of these (see Table 1) can replicate size-frequency observations (Fig 1), but require substantial *ad hoc* assumptions to do so; for instance, in M3 a log-normal antecedent size distribution is needed to create a log-normal distribution of observed sizes (i.e., [M3a]). So this preliminary exploration is detailed in Appendix A, with statistical models incorporating probabilistic growth [M6-11] focussed on below.

**Table 1:** Attributes of the models. Grey shading indicates the variable changed in each group of models. See Section 3 for a discussion of the conceptual framework, which outlines the different parts that comprise the models. SI and WT in column 1 refer to the ‘Stochastic Instability’ and ‘Waiting Time’ models, respectively. Models 1–5 are in Appendix A. The distribution shapes each model can produce are described in sections where they are developed, and acceptable approximations to observations are log-normal, gamma or exponential above the mode.

#	Growth Rate ‘law’			Initial sizes			Growth Style			Growth initiation timing				Can explain size-frequency observations?
	Linear	Exp.	Any	Dirac (i.e., same)	Uniform	Log-normal	Det.	Brownian	Poisson	Dirac (i.e., same)	Uniform	Gaussian	Other	
M1			**✓**	**✓**			**✓**			**✓**				**✕**
M2	**✓**				**✓**		**✓**			**✓**				**✕**
M3		**✓**			**✓**		**✓**			**✓**				**✕**
M3a		**✓**				**✓**	**✓**			**✓**				**✓**
M4	**✓**			**✓**			**✓**				**✓**			**✕**
M4a	**✓**			**✓**			**✓**						**✓**	**✓**
M5		**✓**		**✓**			**✓**				**✓**			**✕**
M5a		**✓**		**✓**			**✓**					**✓**		**✓**
M6	**✓**			**✓**				**✓**		**✓**				**✕**
M7 (SI)		**✓**		**✓**				**✓**		**✓**	i.e., for M6-11 conditions for growth of the flow-set start at a single point in time			**✓**
M8	**✓**			**✓**					**✓**	**✓**				Tail; not roll-over
M9		**✓**		**✓**					**✓**	**✓**				**✕**
M10 (WT)	**✓**			**✓**					**✓**	**✓**				**✓**
M11		**✓**		**✓**					**✓**	**✓**				**✕**

If ice-sediment-water interaction leading to bedform growth is fundamentally stochastic, as proposed by the conceptual model of Hillier et al. [19], then stochastic mathematical models (e.g., [88,89]) may be constructed to formalise variants on this idea. Of possible types of time-series (i.e., temporal) randomness (e.g., [90]), the two most standard and well-established descriptions (e.g., [91]) are selected to create simple stochastic models. Models are therefore created based on ‘white noise’ (Brownian motion) [M6 and M7], developing that of Fowler et al. [22], and Poisson randomness [M8 to M11] as seen in natural processes such as storms impacting land [92]. Particular attention was paid to variants capable of generating distributions that have previously been fitted as approximations to the size-frequency observations (i.e., exponential, gamma, log-normal e.g., [19,22]).

The models employ statistical derivations from texts such as Soong [93], but also use elements from stochastic processes and stochastic differential equations (e.g., [88,94]). All analytical solutions have been validated with pertinent Monte Carlo simulations utilizing 10,000 samples compatible with the statistics of the random quantities (e.g., [95]).

### 5.1. Brownian motion randomness [M6 and M7]

**Fig 5:**
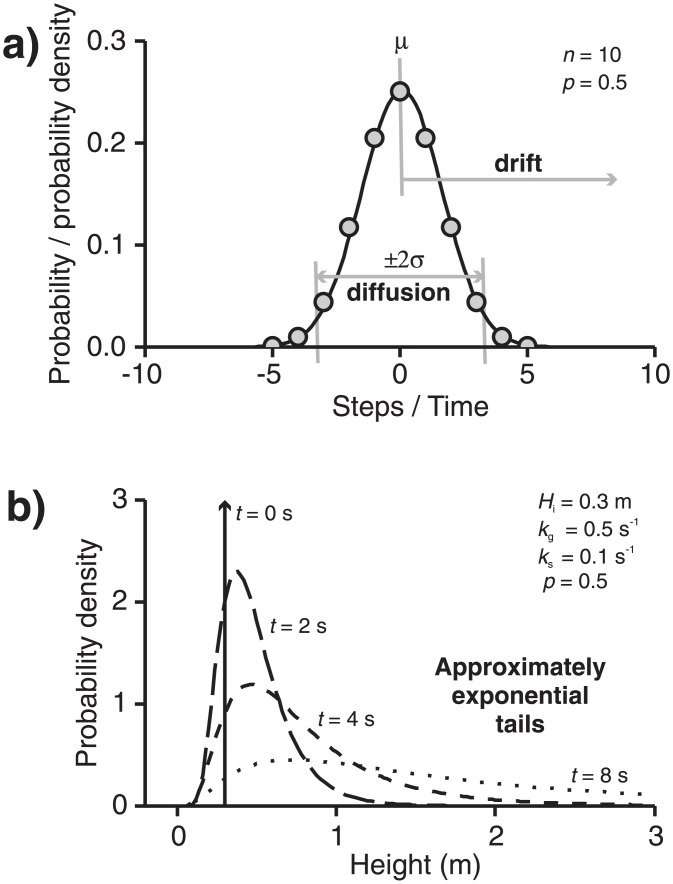
Visualisation of the relationship between a random walk, a Wiener process, and the evolving log-normal size-frequency distribution expected of bedforms in the SI model [M7]. a) Probabilities for the number of discrete steps taken in a random walk (grey circles) are distributed binomially. From Wiener’s work whatever small step length is chosen these are well approximated by normal distribution (black line) of μ = 0 and σ^2^ = *t* i.e., net time spent growing is a normally distributed random variable. If *H* ∝ exp(*t*_*N*_) this defines a log-normal distribution for *H*. b) Height distributions evolving through the SI model [M7] as time increases for some illustrative constants.

Models M6 and M7 incorporate probabilistic growth governed by randomness of a type known by a number of names including ‘Brownian motion’, ‘white noise’, or a ‘1D random walk’ (e.g., [94]). This latter can be pictured as a drunkard in a long, thin alleyway, who either stumbles ‘forward’ or ‘back’ randomly, leading to a distribution of positions that expands with time. If each drunken step takes 1 unit of time, then the net time travelling forward will evolve exactly as distance does, starting to differ increasingly with time, spreading out or dispersing when plotted with predictable statistics: namely, a mean of μ and standard deviation of σ (Fig 5a). Analogously, if changes to a bedform continuously fluctuate between two states (i.e., growth, g, or shrinking, s) in an manner analogous to a random walk (Fig 4c) then net time spent growing (i.e., *t*_N_(*t*) = Σ*t*_*g*_ − Σ*t*_*s*_) is a random variable with a ‘diffusive’ part caused by random motions that is a Gaussian or ‘normal’ distribution [94,96]. Specifically, as the size of steps tend to zero, this is described by a Wiener process denoted *W(t)* [88,94,97,98] and the Gaussian distribution has mean (*μ*) of 0 and variance (σ^2^) of t i.e.,~*N*(0,*t*). Namely, *E*[*W*(*t*)] = 0 and *E*[*W*^2^(*t*)] = *t* with the property *W*(*t*) − *W*(*s*)~*N*(0,*t* − *s*) for *t* > *s* ≥ 0. Statistical ‘drift’ (ξ) where the mean of the distribution increases or decreases with time (*μ = ξt*) can also be accounted for (e.g., [98] p462]; this can be driven by growth being more probable, namely the probability of growing (*p*) being greater than 0.5. This would represent a drunkard capable of some ability to travel forward. Thus, the distribution of *t*_*N*_(*t*) is given by Eq 6 and illustrated in Fig 5a as a hump that both moves or ‘drifts’ and spreads out or ‘diffuses’.

$$t_{N}\left(t\right)=\prime \text{drift}\prime \;+\;\prime \text{diffusion}\prime \;=\xi t+W\left(t\right)$$ (6)

Alternatively, the distribution of *t*_*N*_ created by a Wiener process with drift can be described by a stochastic differential equation (SDE) (e.g., [88,99]) (Eq 7), which integrates to Eq 6 under the initial condition that growth starts at *t*_i_, namely *t*_*N*_(*t*_*i*_) = 0; note that this simple case can be integrated directly since the integral of d*W(t)* is *W(t)* by definition, and it is not necessary to use Itô’s formula. The pdf obtained by either means is more fully expressed by writing out the equation of a Gaussian (Eq 8) with appropriate values of the mean (μ) and variance (*σ*^2^) given by Eqs 9 and 10.

$$\text{d}t_{N}\left(t\right)=\xi \text{d}t+\text{d}W\left(t\right)$$ (7)

$$f\left(t_{N}\right)=\frac{1}{\sigma \sqrt{2\pi }}\text{exp}\left[-\frac{1}{2}\frac{{\left(t_{N}-\mu \right)}^{2}}{\sigma^{2}}\right],\;-\left(t_{\text{f}}-t_{\text{i}}\right)\leq t_{N}\leq \left(t_{\text{f}}-t_{\text{i}}\right)$$ (8)

$$\mu =\xi \left(t_{\text{f}}-t_{\text{i}}\right)$$ (9)

$$\sigma^{2}=\left(t_{\text{f}}-t_{\text{i}}\right)$$ (10)

Statistical drift (ξ) caused by varying *p* is given by *ξ = 2p* − 1. This affects the mean of *t*_*N*_, giving an expression for *μ* as in Eq 11. Two special cases illustrate this behaviour. Without any directional bias, namely if probability of growing and shrinking are equal with *p* = 0.5, *ξ* = 0 and no drift occurs. If all steps are in one direction, namely *p* = 0 or 1, then there is no randomness and *ξ* = ±1 as is appropriate to set growth or shrinkage to a single deterministic rate. However, in the limiting case of *ξ* = ±1 the distribution of *t*_*N*_ cannot diffuse and spread into a Gaussian, and so the spread (i.e., variance) of *t*_*N*_ is also demonstrably affected by *p*, especially near its limits of 0 and 1. This effect is described through well-established results; the discrete Binomial distribution (*n*,*p*) is approximated as a Normal distribution (μ,σ^2^), where *σ*^2^ = *np*(1 − *p*) as n→∞ (e.g., [84] and Fig 5a). Thus, the variance of *t*_*N*_ in Eq 8 is given by Eq 12, where the factor of 4 arises because the step size is doubled, namely (-1,+1) in time versus (0,+1) for the Binomial, which is squared in its impact upon the variance of a random variable (e.g., [93] p81].

$$\mu =\left(2p-1\right)\left(t_{\text{f}}-t_{\text{i}}\right)$$ (11)

$$\sigma^{2}=4\left[p\left(1-p\right)\right]\left(t_{\text{f}}-t_{\text{i}}\right)$$ (12)

Now, it is possible to convert back from time to height, choosing whatever growth law is desired. Firstly, recognising that (*t*_*f*_ − *t*_*i*_) in Eqs 3 and 5 is simply a specific case of net time spent growing (i.e., *t*_N_ = Σ*t*_g_ − Σ*t*_s_), equations for linear and exponential growth can be re-written as in Eqs 13 and 14, respectively. Then, *t*_*N*_ generated by Brownian motion randomness from Eq 8 can be applied to the different growth rates by transformations of the random variables (e.g., Ch 5 of [93]) as in the simpler models in Appendix A (e.g., using Eq 29).

$$H_{\text{f}}=H_{i}+kt_{N}$$ (13)

$$H_{\text{f}}=H_{\text{i}}e^{kt_{N}}$$ (14)

First, consider growth that is linear with time (Eq 13). This is denoted as model M6. The overall amount of time spent growing (*t*_N_) is normally distributed. Since *H*_*f*_ is a simple multiple of this, it will also be normally distributed. As above, analytically determining the pdf of *H*_f_ given the pdf of *t*_N_ is a relatively straightforward task using the standard transformation relationship. This yields Eqs 15 to 17, which describe *H*_f_ as a Gaussian drifting and diffusing as time passes; i.e., not gamma, exponential or log-normal.

$$f_{H_{f}}\left(h_{f}\right)=\frac{1}{\sigma \sqrt{2\pi }}\text{exp}\left[-\frac{1}{2}\frac{{\left(h_{f}-\mu \right)}^{2}}{\sigma^{2}}\right],\;H_{i}-k\left(t_{\text{f}}-t_{\text{i}}\right)\leq h_{f}\leq H_{i}+k\left(t_{\text{f}}-t_{\text{i}}\right)$$ (15)

$$\mu =H_{i}+k\left(2p-1\right)\left(t_{\text{f}}-t_{\text{i}}\right)$$ (16)

$$\sigma^{2}=k^{2}4\left[p\left(1-p\right)\right]\left(t_{\text{f}}-t_{\text{i}}\right)$$ (17)

In contrast, model M7 is formulated for growth that is exponential (Eq 14). Since *t*_N_ is normally distributed, *H*_*f*_ will be log-normally distributed by definition (see Appendix A.3 ‘Variable initiation times’). This is to say that where future increase in a variable is linearly dependent on past progress (i.e., instability, Eqs 4 or 14) a log-normal distribution is produced (e.g., [25]) (Eqs 18 to 20). This assertion can be verified by analytically determining the pdf of *H*_f_ in Eq 14 given the pdf of *t*_N_ and by using the transformation relationship for random variables. Alternatively, the same result can be reached using Stochastic Differential Equations (SDEs). Indeed the form of the result using SDEs is very well established and is known as the solution of ‘*Geometric Brownian Motion*’, which is used for purposes such as predicting stock prices (e.g., [98,100,101]). It is important to note for comparisons, however, that common treatments using SDEs do not allow *p* to vary from 0.5 and, instead of *k*, usually use as their growth constant the effective stochastic equivalent growth rate which for *p* = 0.5 is $\bar{k}=\xi +k^{2}/2$ (e.g. [101] p546).

$$f_{H_{f}}\left(h_{f}\right)=\frac{1}{\sigma h_{f}\sqrt{2\pi }}\text{exp}\left[-\frac{1}{2}\frac{{\left(\text{ln(}h_{f})-\mu \right)}^{2}}{\sigma^{2}}\right],\;H_{i}e^{-k\left(t_{\text{f}}-t_{\text{i}}\right)}\leq h_{f}\leq H_{i}e^{k\left(t_{\text{f}}-t_{\text{i}}\right)}$$ (18)

$$\mu =\text{ln}\left(H_{\text{i}}\right)+k\left(2p-1\right)\left(t_{\text{f}}-t_{\text{i}}\right)$$ (19)

$$\sigma^{2}=k^{2}4\left[p\left(1-p\right)\right]\left(t_{\text{f}}-t_{\text{i}}\right)$$ (20)

**Fig 6:**
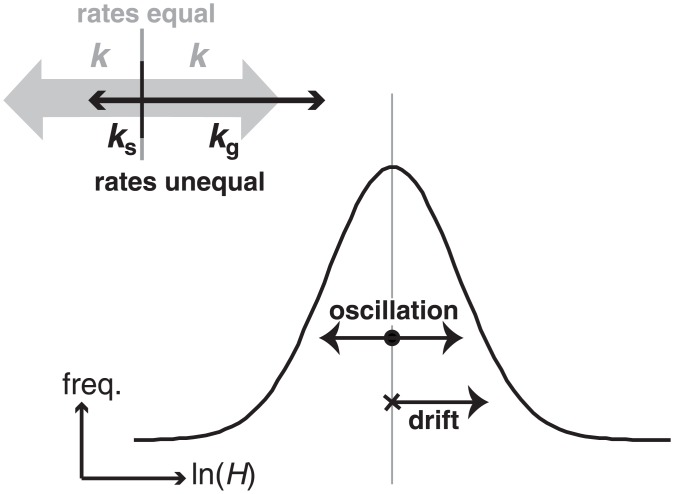
Illustration of how, conceptually, unequal rates of growth and shrinking may be decomposed into components. The components represent: i) oscillation around the centre of a distribution of the logarithm of sizes; and ii) drift of the distribution.

It is now possible to consider another factor that may drive statistical drift of the size distribution in these models: differential rates of growth and shrinking, denoted *k*_g_ and *k*_s_, respectively. The influence of differential rates of growth upon μ and σ is more readily understood if *k*_*g*_ and *k*_*g*_ are re-framed into the drift of the size-frequency distribution and oscillations about the centre of the distribution (Fig 6). The oscillatory component is *k*_av_ = (*k*_g_+*k*_s_)/2, the average rate with respect to the centre of the distribution, and the drift component is *k*_net_ = (*k*_g_+*k*_s_)/2, the imbalance in rates. The oscillations behave exactly as they do for a stationary distribution; so *k* becomes *k*_*av*_ in the equations above. Drift induced this way purely displaces the distribution, and so only affects μ, adding a term so as to cause it to increase at a constant rate with time. Eqs 21 and 22 therefore describe a model [M7] combining Brownian motion randomness in growth with an exponential growth rate that includes the potential for overall growth of the population to be driven by both different probabilities and/or rates of growth and shrinkage; we term M7 the *‘stochastic instability*’ (SI) model. With shrinking forbidden (*k*_s_ = 0) and conceptualised in terms of discrete events, this simplifies to the model of Fowler et al. [22], which dealt with random uni-directional equally sized steps at a single rate creating growth.

$$\mu =\text{ln}\left(H_{\text{i}}\right)+t\left[k_{\text{net}}+\left(2p-1\right)k_{\text{av}}\right]$$ (21)

$$\sigma^{2}=k_{\text{av}}^{2}4\left[p\left(1-p\right)\right]t$$ (22)

Values for μ and σ of the SI model [M7] may readily be estimated (see Appendix B) directly from mapped bedform sizes (e.g., Fig 1). Through Eqs 21 and 22 the SI model therefore predicts trajectories of characteristics of the observed size distribution (μ_obs_ and σ_obs_) through time; specifically μ_obs_ is expected to be proportional to the square of σ_obs_.

It is also possible to make predictions about the size differences (e.g., Δ*H*) of flow-sets of bedforms across an observational window (i.e., at *t*_1_ and *t*_2_). First, all bedforms should be active and change size, and there should be a mixture of shrinking and growing. Secondly, in spite of the scatter caused by randomness, Δ*H* should relate to *H* (Eq 4). Thirdly, by the definition of a diffusive Wiener process *t*_*N*_ in any time period is normally distributed, and thus the distribution of the differences in height Δ*H* should be log-normal. Furthermore, since the time difference is known, parameters of the SI [M7] model (i.e., *p* or *k*_net_, *k*_av_, total duration of growth period) may be uniquely constrained (Table 2).

**Table 2:** Table of testable predictions for the WT [M10] and SI [M7] models.

	Characteristic	Expectation: WT model [M10]	Expectation: SI model [M7]	Test/Investigative method
1	Size-frequency distribution	Gamma; through time or across Δ*t*. *β* constant; *α* ∝ *t*	Log-normal through time or across Δ*t*. *μ* ∝ σ^2^ ∝ *t*	Repeat survey under active ice, or plot palaeo-forms from multiple flow sets (e.g., *μ*_obs_ vs *σ*_obs_)
2	Spatial pattern of ice flow variables or conditions	Poisson fluctuations in time, at least at a bedform scale	Constantly fluctuating, at least at the spatio-temporal scale of bedform genesis	Estimate basal ice conditions using geophysics or invert for them from satellite observations of the ice surface (e.g., [6])
3	Fraction shrinking vs growing	All active forms grow (i.e., Δ*H* is +ve)	All active. Δ*H* a mixture of growing and shrinking; fraction *p* growing.	Repeat survey under active ice; e.g., repeat [13]
4	Growth rate	Constant. With Δ*t* known, Δ*α* and Δ*β* are constrained and so are *λ* and *k* (Eq 26), so overall time to create flow set also deducible.	Exponential, i.e., proportional to *H*. If Δ*t* known, Δ*μ* and Δ*σ* and so *p* or *k*_net_ and *k*_av_ are constrained (Eqs 21 and 22), so overall time to create flow set also deducible.	Repeat survey under active ice.
5	Fraction unchanged	>0 for small Δ*t*	Small; depends on definition of change	Repeat survey under active ice.

### 5.2. Waiting time randomness [M8 to M11]

In contrast to Brownian motion randomness, there is another well-established type of temporal randomness called Poisson randomness (e.g., [94]). This is investigated in models M8 to M11.

In ‘Poisson’ randomness, the gaps between events that occur randomly at a given rate (λ, number per unit time) are distributed according to the exponential or ‘*waiting time*’ distribution (e.g. [97] p39-40). This distribution is, for instance, used to model the times between shoppers arriving at a supermarket checkout. So, if the arrival or ‘event’ is the change in state (i.e., growth to inactivity) of a continuous process (cf. [91]) it also describes inter-event periods in which bedforms may grow (Fig 4b). Thus, if only a single episode of growth (e.g., the last) is preserved, net time spent growing (*t*_N_) is distributed according to an exponential distribution (Eq 23).

$$f_{T_{N}}\left(t_{N}\right)=\lambda e^{-\lambda t_{N}},\;t_{N}>0$$ (23)

As in Section 5.1, this is formulated in terms of time spent growing so that any desired growth rate law can be readily applied to determine distributions for *H*_*f*_. The distributions of *H*_*f*_ that are generated by taking *t*_N_ as a random variable can be deduced by transformations of random variables as above (e.g., Ch 5 of [93]).

Consider first model M8, in which growth is constant with time (Eq 1). With *t*_N_ as above, an exponential distribution of heights results (Eq 24). This, however, is not so for exponential growth (Eq 14) in model M9. This produces a distribution that is not exponential, log-normal or Gamma. M8 predicts that the exponent of the tail of the observed pdf of final heights (*H*_f_) is *λ*/*k* as in Eq 24, where growth rate (*k*) is from Eq 13. This exponent is readily estimated from mapped sizes [19], and is not expected to progress with time. It is predicted to be set by, vary in equilibrium with, and therefore reflect formative (i.e. ice or water) flow conditions.

$$f_{H_{\text{f}}}\left(h_{\text{f}}\right)=\frac{\lambda }{k}e^{-\lambda \left(\frac{h_{\text{f}}-H_{\text{i}}}{k}\right)},\;h_{\text{f}}>H_{\text{i}}$$ (24)

However, instead of being in equilibrium with flow, glacial bedforms may be in a transient state with respect to flow. This is incorporated within models M10 and M11. If bedforms are created by a number (*n*_b_), on average, of building episodes then *t*_N_ is the sum of *n*_b_ exponential distributions; this is a two-parameter Gamma distribution denoted *t*_*N*_ ~ Γ(*α*,*β*) [84]. The Poisson rate (λ) as defined above is now standardly denoted β and is the ‘rate parameter’ of the Gamma distribution. The shape parameter of the Gamma distribution (α) is simply equal to *n*_b_ (e.g. [97] p292). On average in M10 and M11 the number of building episodes is a multiplication of the rate at which they occur and the time that has elapsed, namely *n*_b_ = 0.5*λt*, which is illustrated in Fig 4b. The factor of 0.5 arises because two switches (‘on’ and ‘off’) are needed for each growth period.

The distributions of *H*_*f*_ that are generated in these Poisson multi-event models [M10 and M11] can be deduced by taking *t*_N_ as a Gamma distributed random variable, using growth rates in equations Eqs 13 and 14, and as in previous sections then using transformations of random variables (i.e., Eq 29). M10 has constant growth (Eq 13), we term it the *‘waiting time*’ (WT) model, and a Gamma distribution of heights results. This is not so for exponential growth (Eq 14) upon which model M11 is based, which produces size distributions that are neither log-normal or Gamma.

The parameters of the WT [M10] model (i.e., λ, *k*, and t) may be constrained from the rate (β) and shape (α) parameters of the final height distributions (*H*_f_). They are related as in Eqs 25 and 26. Observed values are denoted β_obs_ and α_obs_, are readily estimated (e.g., figure 1 of [19]), and are predicted to be constant and increase linearly with time respectively.

β=λ/k (25)

$$\alpha =n_{\text{b}}=0.5\lambda \left(t_{\text{f}}-t_{\text{i}}\right)$$ (26)

It is possible to make predictions about the size differences (e.g., Δ*H*) expected across a time window (i.e., at *t*_1_ and *t*_2_). First, all bedforms that have changed should have grown, and a fraction should not have changed if the number of building events (*n*_b_ = α) is small. Secondly, growth should be at a constant rate and Δ*H* should not correlate strongly with *H* (Eq 1). Thirdly, the ‘memoryless’ nature of the Poisson process dictates that Δ*H* should be a Gamma distribution. Furthermore, since the time difference is known, the rate constant of bedform growth (λ) could then be estimated uniquely through the two observations of α (i.e.,Δ*α*_obs_ = *α*_2_ − *α*_1_ = 0.5*λ*Δ*t*). Then, growth rate (*k*) could be calculated through either observation of β (see Table 2).

## 6. Results

The right hand column of Table 1 lists which models produce size-frequency distributions that have been argued to reasonably approximate mapped observations (i.e., log-normal [22,35,36], gamma, or exponential above mode [19]). Fig 1 shows a direct comparison, illustrating how well each of these three alternatives fit the data: solid line is an exponential distribution, generated by model M8; dashed line is a log-normal distribution generated by M7, the Stochastic Instability (SI) model; dotted line is a gamma distribution generated by M10 the Waiting Time (WT) model. Other models, however, can fit. By invoking substantial *ad hoc* assumptions (see Appendix A), some models that do not involve stochasticity in growth through time [M3a, M4a, M5a] can also replicate size-frequency observations. Fig 2 and Appendix A also demonstrate some of the shapes generated by the other models. It is important to note that fitting statistical distributions as in Fig 1 in itself leads to parameters (e.g., β and ρ, or ϕ and λ) that are only descriptive empirical quantities; it is the statistical bedform growth models that relate the parameters to key aspects of the physical process: antecedent topography, growth rate (e.g., exponential), and the timing of growth.

## 7. Discussion

To gain additional insight into the plausibility of conceptual models of the growth of subglacial bedforms, this paper takes well-established statistical behaviours (e.g., types of temporal randomness) and integrates them with plausible growth rate behaviours (e.g., [20]) to explore which combine to produce reasonable approximations of the observed size-frequency distribution of subglacial bedforms (i.e., exponential, Gamma, or log-normal (e.g., [19,22])). Exactly as any model (e.g., numerical ice sheet models) these contain approximations and assumptions, but are constructed to capture key aspects of the physical processes in order that these might be evaluated by comparing modelled outputs to observations. In 7.1, the statistical models [M1-M11] are evaluated in terms of their ability to explain i) the size-frequency observations whilst invoking the least number of *ad hoc* or arbitrary assumptions, ii) their internal consistency, and iii) their ability to explain all other relevant observations (e.g., geophysics). The implications of the favoured model are then discussed (section 7.2), followed by some suggestions for future work (section 7.3).

### 7.1 Evaluation of the models

The simplest models created [M1-5] do not involve stochasticity in growth through time. For any of these (see Table 1) to replicate size-frequency observations (Fig 1) they require substantial *ad hoc* assumptions or special pleading, discussed in Appendix A. This we interpret as making these models, as constructed, less plausible and giving some weight to the view that neither ‘classic’ deterministic growth nor antecedent bedform-scale topography are sufficient to explain bedform sizes. It should be noted, however, that the failure of one particular modelling realisation of an envisaged process rarely excludes that process.

Models M6 to M11 follow up on the conceptual model of [19] in that they are based on variations in growth through time. Constructions M6 and M9 do not match the size-frequency observations (Table 1) and they can be ruled out. M8 can reproduce the exponential tail, but to allow it to fit the data fully it must either invoke selective post-formational degradation or an argument that observational data have missed most small bedforms in order to create the roll-over. This is debatable; first, even the ~25% recovery rate affecting small drumlins is insufficient to wholly explain the roll-over in the UK data [31,102], and second the very many small forms expected of an exponential distribution are mapped in high-resolution data of neither previously glaciated (e.g., [103]) nor recently uncovered [40] drumlin fields. In contrast to M8, both types of temporal randomness, when combined with appropriate growth rates into the SI and WT models (i.e., in M7 and M10, but not M6 or M11), fit the widespread palaeo-bedform size data. Neither Poission nor Brownian Motion randomness in growth have yet been specifically identified under active ice, but they have been observed commonly in natural processes including bedform evolution [25–28,30,57,80,92,96], and so are supported by analogy. This, we argue, makes their introduction significantly less *ad hoc* than the arbitrary assumption of convenient statistical distributions in M3a to M5a. Note, for instance, that the temporal variation that distributes *t*_N_ in the SI model [M7] intrinsically creates the Gaussian distribution arbitrarily invoked by M5a.

**Fig 7:**
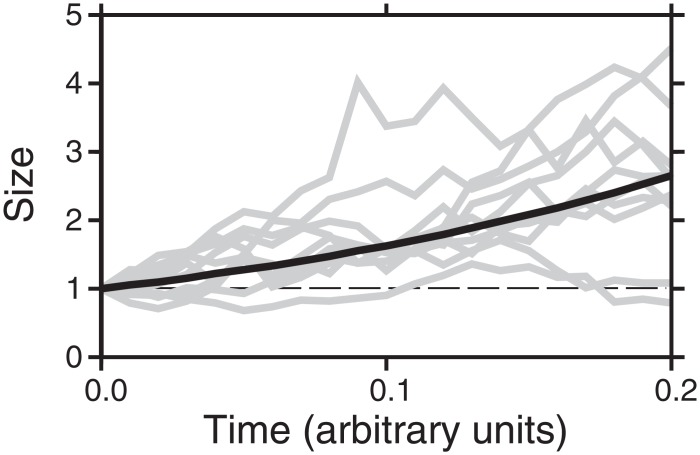
Evolution of bedforms including randomness through time. The evolution of sizes of ten illustrative bedforms including randomness in their growth through time (grey lines). These differ from a deterministic path (black line). For a sufficiently large number of bedforms, the average properties (e.g., mean size) of a flow set closely approximate the deterministic path. Bedforms are ‘born’, last pass a threshold minimum observable height (e.g., 1 unit, dashed line), at different times.

Significantly, and in their favour, models M7 (‘stochastic instability’: SI) and M10 (‘waiting time’: WT) also explain other independent observations of bedforms without any further *ad hoc* additions. First, probabilistic growth decouples initial and final sizes, allowing the intervening physical process to dominate the characteristics of the ultimate size-frequency distribution; that is, illustratively, the randomness in growth shown in Fig 7 dictates the size-distribution, not the initial size. This offers an explanation for the observation that drumlins with their typical size-distribution can originate irrespective of differences in environment (e.g., till/bedrock lithology) [42,43]. Secondly, the observed structure (e.g., internal stratigraphy (e.g., [12,40])), the variety of composition (e.g., [42,43]), and the substantial (e.g., ±50%) scatter in the sizes and elongations commonly seen for proximal palaeo-forms within a flow-set (e.g., [16,39,45,104]), might be expected to result from randomness and fluctuations in characteristics of the ice-sediment-water system in space and time. By their design, the WT and SI models are also consistent with the geophysical, remotely sensed, and sedimentological evidence for spatio-temporal variability in ice flow velocity and the bed beneath ice sheets, which was outlined in sections 3.1 and 3.2. Thus, the widespread dataset of palaeo-bedform sizes points towards a view where ice-water-sediment dynamics (i.e., change through time) likely has a fundamentally random element that physics-based models of bedform genesis could usefully incorporate; to date, some models have been seeded with initial random height perturbations [48,79], but what if any temporal randomness to emerge from this has not been explicitly examined. Fowler et al. [22] demonstrated that a statistical model can reconcile observations with the hypothesis of Hillier et al. [19], but the variety of statistical models considered here allows us for the first time to distinguish process dynamics (i.e., randomness through time) as the most plausible origin for the necessary variability out of the main candidates.

It is possible to argue that one type of bedform-scale dynamics is more likely, i.e., differentiate between the SI [M7] and WT [M10] models. First, by visual inspection the log-normal shape produced by the SI model arguably fits the size-frequency data than the gamma distribution of the WT model, especially for *L* and *W*, and for small sizes (see Fig 1). Secondly, it allows bedforms to shrink as seems probable from the geophysical observations [11,12], which the WT model does not. Thirdly, the SI and WT models may also be evaluated through their internal consistency between observations for the three dimensions *H*, *W*, and *L*. Taking the simplest assumption that all dimensions change size together (i.e., *t* and *p* are the same), Eq 22 can be used to constrain relative growth rates (e.g.,*k*_*avH*_/*k*_*avW*_) for the dimensions within the SI model (Eq 27). Values for σ calculated for mapped UK drumlin data given in Fig 1 then indicate that increasing *H* is the primary mode in their genesis, namely its growth rate constant is greatest (*k*_*avH*_ > *k*_*avL*_ > *k*_*avW*_). This is plausible. In contrast, using Eq 26, α values for the WT model [M10] imply a different number of growth episodes for each dimension. This is less easily explicable. Thus, with these factors taken together, we choose to favour the SI model over the WT model.

$$\frac{\sigma_{L}}{\sigma_{W}}=\frac{k_{avL}}{k_{avW}},\;\;\;\;\frac{\sigma_{H}}{\sigma_{W}}=\frac{k_{avH}}{k_{avW}},\;\;\;\;\;\;\frac{\sigma_{L}}{\sigma_{H}}=\frac{k_{avL}}{k_{avH}}$$ (27)

Alternatively, stochasticity in the ice-sediment-water system may differ from the Brownian motion of our SI model, but with exponential growth still produce log-normal size-frequency distributions because of the central limit theorem (CLT) [22]. Fowler et al. [22] interpret this as favouring growth through discrete 'events' of constant size, but the CLT has other interpretations (e.g. p88 of [105], p266 of [106]), so this is not necessarily required. For instance, if growth of each bedform is governed by discrete ‘events’ of random size, selected from any frequency distribution, the CLT predicts a log-normal distribution of sizes in a flow set. Similarly, if bedforms grow by many growth periods of a random duration selected from any frequency distribution, the CLT dictates that effective *t*_N_ will be Gaussian as required. However, even given this, the SI model is still likely to be a useful *empirical approximation*. If the factors dictating bedform-scale randomness (e.g., supra-glacial lake drainage patterns) relate to broader ice-sediment-water conditions then parameters fitted as for the SI model (i.e., μ, σ) will still provide a useful statistical link between observations at the flow-set level and theory such as in numerical ice flow models (e.g., by plotting spatial distributions).

### 7.2 Implications of the SI approximation

The SI model, if it is to be accepted as most likely, has a number of implications. Bedforms are expected to change size randomly through time in a manner approximating Brownian motion, growing on average exponentially (Fig 7). The quantitative, observable corollaries of this are listed in Table 2. A number of points, however, need some further explanation.

First, the SI model implies that it is not necessary to invoke a lower ‘physical threshold’ on drumlin length or width [16] or an upper limit for *H* a quenching (a.k.a. ‘capping’) mechanism to limit their upper ‘critical size’ (e.g., [20,77,78,107]). In the SI model very small sizes are simply less likely and no lower threshold is needed. As an alternative explanation for the absence of extremely large bedforms, the SI model and its simpler variant (i.e., [22]) must invoke growth that is ‘transient’, namely that it occurs within a time window of limited duration. Simply, insufficient time has passed for very large forms to be created. Observations of active bedforms do not yet indicate which means of limiting the largest sizes is most plausible, but several mechanisms can be imagined that allow growth periods forming flow sets to be of limited duration. In a steady-state view, meso-scale patches of bedforms could be periodically flattened by conditions adverse to the existence of bedforms. Alternatively, favourable patches may only occur transiently (e.g., [39]) or time-transgressively (e.g., [38]) as ice sheets melt and retreat. However, to explain bedform prevalence, these mechanisms must commonly occur. Size-frequency observations give two tentative indications that a time limitation (e.g., SI model) affects glacial bedforms rather than a physical cap in an equilibrium model (e.g., [78]). The first indication is that fluvial bedforms measured at equilibrium with flow do not have a log-normal distribution, but one that peaks at larger sizes (figure 6a of [26]) as if sizes where tending to bunch below some fuzzy threshold. The second indication is that if glacial bedforms were to grow and then to ‘freeze’ [78] at a sharp upper limit a peak in frequencies would be expected, but this is not observed in Fig 1c (i.e., at 34 m).

Secondly, assuming all dimensions change size together (i.e., *t* and *p* are the same), relative growth rates estimated from UK observations (Fig 1, Eq 27) (i.e., *k*_*avL*_ > *k*_*avW*_) indicate that drumlins elongate as they grow (e.g., [16,31]). Note that no relationship between the dimensions was placed into the SI model that might have prescribed this observation. Perhaps they continue into mega-scale glacial lineations (MSGL) as part of a genetically-linked bedform continuum (cf. [108,109]), where *H* and *W* are in equilibrium restricted by stochastic interactions with ice and neighbouring bedforms whilst elongation continues.

Thirdly, Fowler et al. [22] put forward an explanation to demonstrate that size observations do not necessarily falsify the exponential growth hypothesised in the physically-based till ‘instability models’ of bedform genesis (e.g., [20]). Here, a variety of different explanations are considered, and exponential growth still features in the one that is apparently most plausible. Thus, through this comparison, the SI model strengthens the tentative observational support for exponential bedform growth (i.e. by linear instability). On the other hand, from two-parameter fits to observed data collated in a small number of distributions (e.g., Fig 1) it is not possible to distinguish between existing linear instability mechanisms, namely till or heat-flux (e.g., [20,33]). Future work plotting the spatial distribution of parameters (μ, σ) of mapped palaeo-bedforms against numerically modelled predictions of growth rate (*k*) for each mechanism for a past ice sheet could, however, distinguish them. Other possible tests and applications of the SI model are considered below.

### 7.3 Future Work: Testing and applying the SI model

The SI model [M7], if correct, suggests tentative analytical links between parameters fitted to observed size-frequency distributions and ice sheet properties, such as ice velocity; the SI model links size observations (μ, σ) to growth rate *k* (Eqs 21 and 22), which relates to physical parameters (e.g., [33]). Eq 52 of Fowler [110], for instance, related *k* to (*AN*/2*η*)^1/2^ within which *A* is illustratively proportional to ice velocity. Similarly, Shoemaker [56] related *k* to subglacial flood water velocity to a power $\frac{16}{3}$. Thus, predicted relationships (e.g.,$k\propto \sqrt{v}$) can contribute to geomorphological debates such as the interpretation of *L* in terms of *t* or *v* (e.g., [3]). Admittedly, the problem is under-constrained since there are three variables (*p* or *k*_net_, *k*_av_, and *t*) and two observables (μ, σ). If, however, more can be learnt about one of these through direct observation or experimentation (e.g., *p*) the other two (e.g., *t* or *k*) could be determined remotely from a single morphometric analysis.

The SI model makes quantitative predictions that are distinctively different from the WT model or deterministic ones, as detailed in Table 2. This makes it testable and falsifiable by observations from modern subglacial environments. The predictions are, for example, testable by repeating at *t*_2_ a past (i.e., at *t*_1_) geophysical survey under active ice (i.e., [13]). In addition, plots of size-frequency parameters obtained for a number of observed flow sets are diagnostic of different models (see Section 5); for instance, in the SI model *μ* ∝ σ^2^, so plots of *μ* against σ^2^ will display linear trends if *t* varies whilst the other variables are held constant. Plotting spatial variations in parameters could also be an additional constraint upon physics-based models of bedform genesis. Illustratively, consider a numerical model used to estimate ice flow in a past ice sheet (e.g., [111]), a physics-based model of bedform genesis (e.g., [33]), and a hypothesised set of conditions (e.g., based on basal shear stress) for drumlin formation. Then, the modelled ice-sheet conditions set *t* for flow-sets geomorphologically mapped for that ice sheet, and in conjunction with the model of bedform genesis they also set a numerical prediction for *k*. Furthermore, since *t* is constrained in the context of this test, *k* and *p* can be determined for the mapped flow sets by using a statistical model (see above). Thus, through the spatial distribution of *k*, a way exists to quantitatively compare models and observations. Patterns in *k* could either be of absolute or relative values, and *k* and *p* may relate to properties of ice flow (e.g., *v*) or postulated floods depending upon the drumlin formation model selected. In particular, the ability or not to correctly predict the distribution and properties of flow sets may help to further constrain which ice sheet models, or members of an ensemble of potential realisations, is most valid.

Since we do not attempt to develop all possible models here, the wider point is that statistical modelling provides a tool to develop and falsify conceptual models of bedform growth. The same is true for other bedforms where measurement of key processes is challenging (e.g., in-situ on barchan dunes) and where time-series of digital elevation models are becoming available but statistical work is limited (e.g., [18]). With respect to fluvial environments, developing our analytical work could create statistical distributions reflecting underlying mechanics, improving upon existing distributions as descriptors (e.g., [26]) and allowing more to be extracted from field observations.

## 8. Conclusions

The emergence and growth of subglacial bedforms is difficult to observe, significantly limiting our ability to accurately parameterise basal processes beneath ice sheets. In this paper, a novel approach has been taken, developing new probabilistic growth models and comparing their predictions with observed distributions of palaeo-bedform sizes. The variety of explanations both permits a number of models to be discounted and the relative plausibility of the rest to be assessed for the first time. The ‘*stochastic instability’* (SI) model, modified from Fowler et al. [22] and extended to encompass bedforms shrinking, is argued to provide the best fit to observations. Not only does it fit the size observations [22], but it appears to do so with fewest *ad hoc* assumptions whilst being internally self-consistent between metrics (e.g., height and width) and in accord with other observations (e.g., geophysical). Thus, our analysis strengthens a view [19,22] where the ice-sediment-water dynamics and sediment flux have significant elements of randomness in space and time (i.e., not continuous or monotonic) and cause both erosion and deposition. This view is developed to explicitly argue that (i) flow-related processes at the ice-bed interface rather than initial bedform-scale topography govern bedform sizes and (ii) drumlins elongate with time. Furthermore, parameters of mapped size-frequency distributions are explicitly linked with ones related to flow (i.e. ice and water) for the first time, accompanied by an illustration of an avenue for how this may be used to improve calibration of basal conditions in numerical ice sheet models and achieve a better understanding of conditions at the base of ice sheets. Lastly, we demonstrate that it is possible to provide testable, distinctive predictions that will allow models to be distinguished using a hypothesised repeat geophysical survey of bedforms under active ice. Note that none of the work presented here precludes or conflicts with observations of structured spatial patterning in the bedforms.

## Appendix A: Preliminary Exploration

Following the trajectory of work that developed stochastic sub-aerial landscape evolution models to explain topography’s typical fractal statistics [112], this appendix formalises statistically for the first time simple models representing the prevailing ‘classic’ view that bedform growth through time is not random, which has not yet been undertaken for subglacial bedforms. In these simpler models, elements of the potential spectrum of randomness within the proposed meso-scale patches are, effectively, turned off.

The first models [M1-3] represent the more plausible realisations of the ‘classical’ view where bedform growth through time is not random. M1 considers the simplest, entirely deterministic, case. It is possible that the bedform-scale topography prior to bedform creation is not planar, so models M2 and M3 include variability in initial bedform height. It has also been proposed that bedforms are not ‘born’ at the same time (cf. [11,113]), so models M4 and M5 assess the possibility that each bedform could start to grow at a different time. The models are described then evaluated.

### A.1. Entirely deterministic growth [M1]

**Fig 8:**
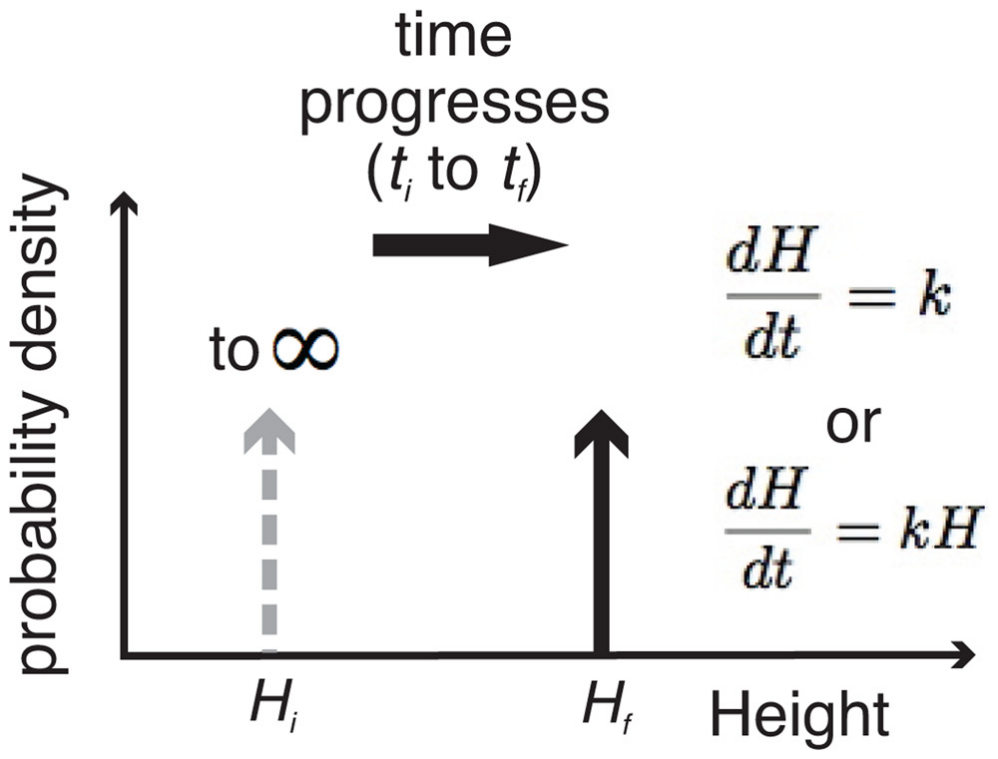
Probability density functions (pdfs) for the simplest model [M1]. In this model drumlins have a single initial height *H*_i_, then grow deterministically through time.

Model M1 considers multiple independent bedforms all of a single initial height (*H*_i_) growing according to any given deterministic mechanism; the ‘classical’ view that has yet to be explicitly tested. The bedforms will all reach the same final height (*H*_f_) as each other after any time has elapsed (i.e., *t*_f_—*t*_i_), whatever their growth rate (Fig 8). This model starts with a Dirac delta function as the pdf (probability density function) of *H*_i_ and produces the same pdf of *H*_f_ at a later instant in time *t*_*f*_, namely a single vertical spike on plots such as Figs 2 or 8.

### A.2. Variable initial topography [M2 and M3]

Models M2 and M3 are designed to give insight into whether or not the observed final size-frequency distribution may simply arise as a result of an inherited distribution of initial sizes, without recourse to stochastic behaviour during growth. These models are stochastic in the initial conditions only; that is, the initial condition of Eq 2 is modelled as a random variable following a prescribed pdf that reflects a chosen initial size distribution.

**Fig 9:**
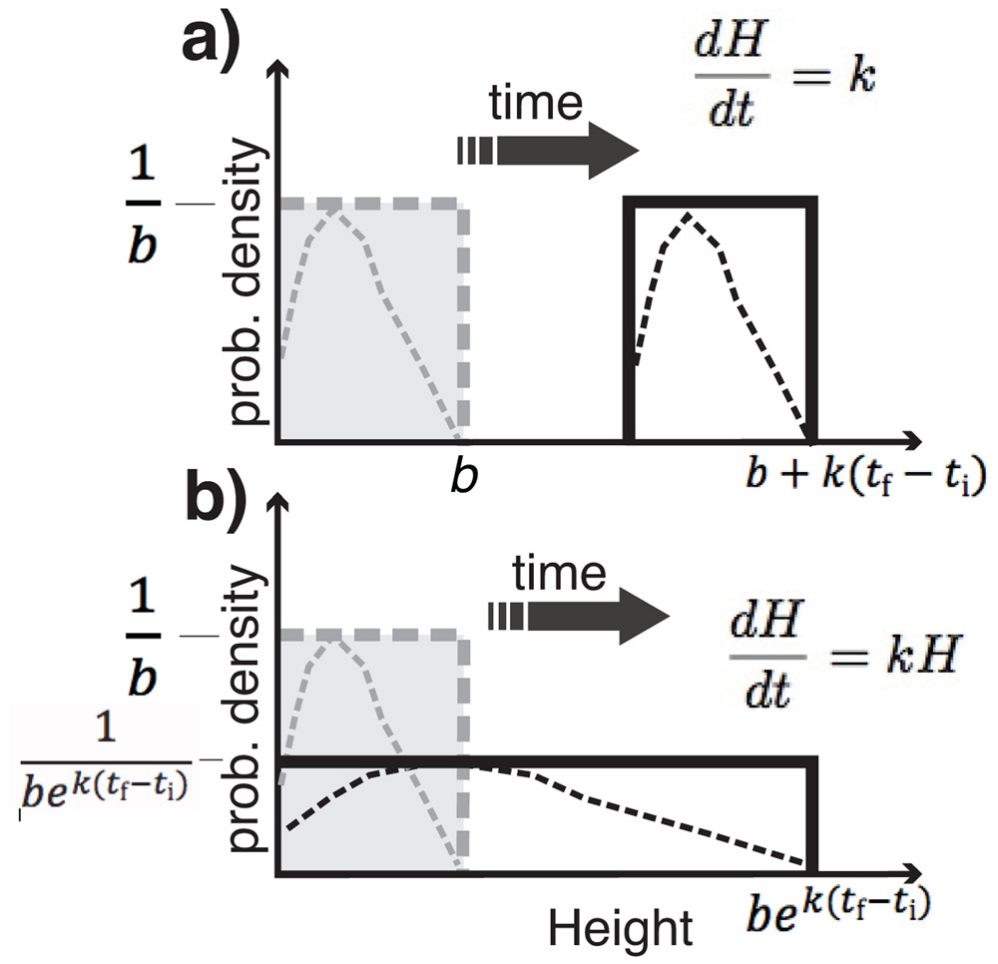
Pdfs for models with deterministic growth and variable initial topography a) linear growth [M2] b) exponential growth [M3]. Initial *H* distribution *H*_i_ (grey, dashed line) changes to the final one *H*_f_ (black outline) as time progresses. Dotted lines are an arbitrary function. Cases shown are where smallest *H*_i_ is zero; *a* = 0.

Proto-bedforms of initial height *H*_i_ follow a uniform distribution, that is they are equally distributed across a range of heights between *a* and *b* (Eq 28), which is the width of the grey boxes on Fig 9, and grow deterministically.

$$f_{H_{\text{i}}}\left(h_{\text{i}}\right)=\{\begin{array}{cc} \frac{1}{b-a}\text{,} & \text{for}\;a<h_{\text{i}}<b\\ 0, & \text{elsewhere} \end{array}$$ (28)

So defined, *H*_i_ is a random variable; thus, since *H*_f_ in Eqs 3 and 5. is a function of *H*_i_, it is also a random variable whose distribution can be determined. Determining the pdf of *H*_f_ given the pdf of *H*_i_ is a relatively straightforward task. To this aim, the standard transformation relationship

$$f_{\text{Y}}\left(y\right)=f_{\text{X}}\left(g^{-1}\left(y\right)\right)\left|\frac{\text{d}}{\text{d}y}g^{-1}\left(y\right)\right|$$ (29)

relating random variables *y* and *x* is invoked assuming a relationship of the form *y* = *g*(*x*) (e.g., Ch 5 of [93]).

If growth is linear with time (Eq 1) [M2], the shape of the initial distribution is not altered (Eq 30) and it moves right as illustrated in Fig 9a. So, if any non-trivial growth (e.g., 4 m) has occurred, it is not possible to construct a pdf for *H*_i_ that still contains low amplitude bedforms; for example, even the smallest initial height of 0 m would have grown to 4 m. For mapped size data the mode (*ϕ*_obs_) would increase linearly with time, but the exponent of the right-hand tail (λ_obs_) [19] would stay constant.

$$f_{H_{\text{f}}}\left(h_{\text{f}}\right)=\{\;\begin{array}{cc} \frac{1}{b-a}\text{,} & \text{for}\;a+k\left(t_{\text{f}}-t_{\text{i}}\right)<h_{\text{f}}<b+k\left(t_{\text{f}}-t_{\text{i}}\right)\\ 0, & \text{elsewhere} \end{array}$$ (30)

If growth is caused by linear instability [M3] (i.e., is exponential as in Eq 4) then the distribution elongates (Eq 31, Fig 9b) but does not alter the relative abundances of different bedform sizes (e.g., 5th, 50th and 95th percentiles of *H*). Indeed, the pdf can be imagined as being drawn on a sheet of elastic material so that, even if it is any arbitrary function (dotted lines), it will be elongated but not otherwise distorted. Thus, to end up with an approximately log-normal distribution as observed for bedforms (e.g., Fig 1), a landscape must start with a log-normal distribution; this *ad hoc* modification of M3 is denoted M3a. For mapped size data M3a would have both *ϕ*_obs_ and 1/λobs increasing linearly proportional to each other and with the duration of the bedform building episode, and this would happen along a trajectory set by the shape of the initial distribution.

$$f_{H_{\text{f}}}\left(h_{\text{f}}\right)=\{\begin{array}{cc} \frac{1}{\left(b-a\right)e^{k\left(t_{\text{f}}-t_{\text{i}}\right)}},\; & \text{for}\;ae^{k\left(t_{\text{f}}-t_{\text{i}}\right)}<h_{\text{f}}<be^{k\left(t_{\text{f}}-t_{\text{i}}\right)}\\ 0, & \text{elsewhere} \end{array}$$ (31)

### A.3. Variable initiation times [M4 and M5]

Models M4 and M5 formalise the glaciological hypothesis in which bedforms are not ‘born’ at the same time and therefore, at any point in time, will have been growing for different durations [11,113]. Proto-bedforms of an initial (constant) size *H*_i_ start growing at times distributed according to a uniform distribution from an earliest time defined as *c*; i.e., a constant number are created per unit time as the building of the flow set progresses. All continue growing until a final, constant time (*t*_f_). The time at which bedforms’ growth starts, *t*_i_, is now a random variable (Eq 32) making final height (*H*_f_) also a random variable since it is a function of *t*_i_. The pdf of *H*_f_ can be determined similarly to the previous section by resorting to the transformation relationship of Eq 29.

$$f_{T_{\text{i}}}\left(t_{\text{i}}\right)=\{\begin{array}{cc} \frac{1}{t_{\text{f}}-c}\text{,} & \text{for}\;c<t_{\text{i}}<t_{\text{f}}\\ 0, & \text{elsewhere} \end{array}$$ (32)

**Fig 10:**
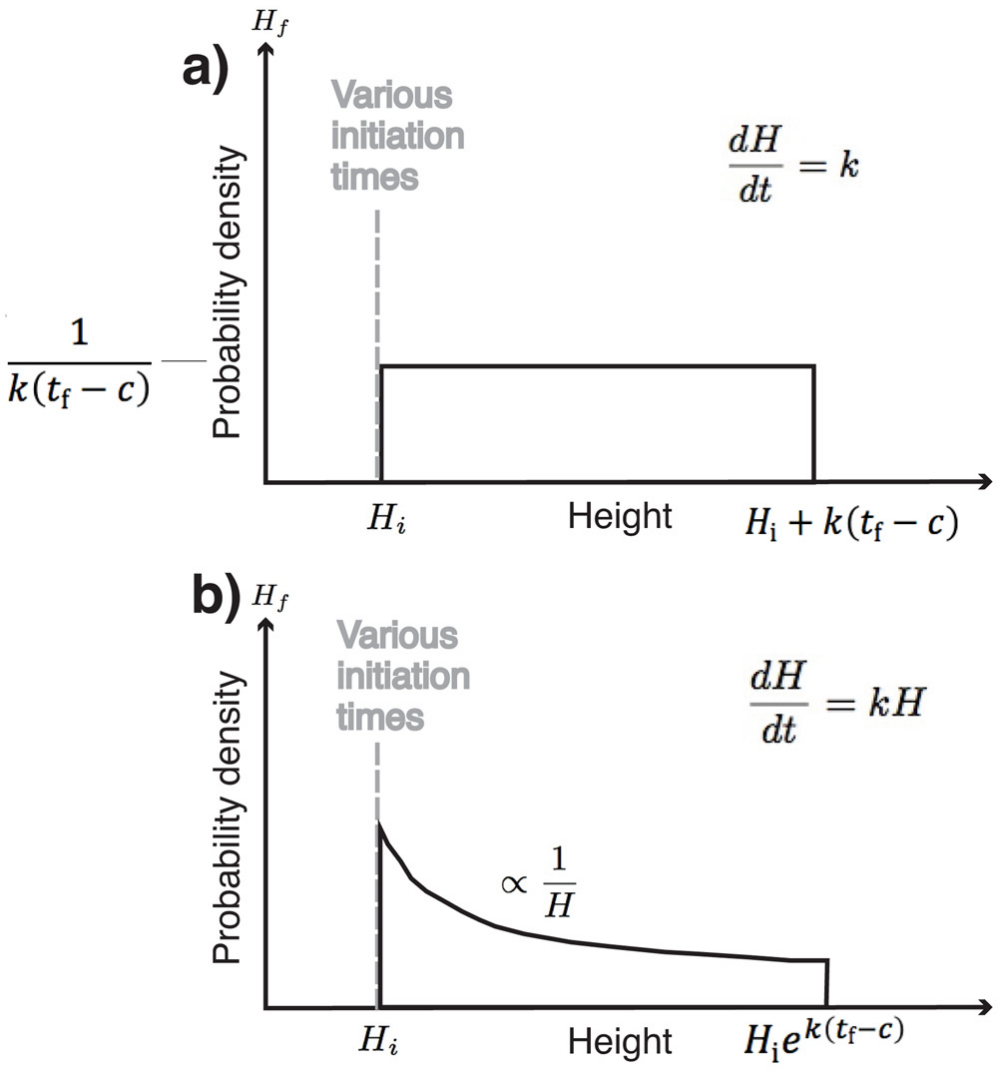
Pdfs for models with deterministic growth where bedforms have constant initial heights, but a uniform distribution of initiation times (i.e., initiation rate is constant through time). a) linear growth [M4] b) exponential growth [M5]. Initial distribution (grey, dashed line) changes to the final one (black outline).

If growth is linear with time (Eq 1) [M4], then a uniform distribution of final heights is produced (Fig 10a, Eq 33). In general, *ad hoc* manipulation of the form of the pdf of *t*_i_ will be directly reflected in the output form of *H*_f_. A linearly increasing production rate (number per unit time), for instance, would produce a linearly decreasing frequency with increasing *H*_f_ because the larger number of recently produced forms have not yet had time to grow. Thus, an approximately Gamma distribution (e.g., Fig 1), for instance, could be created by a production rate that started slowly, built approximately exponentially to a peak and then died rapidly before *t*_f_; this variant is denoted M4a. If interrupted at any point before the distribution was fully formed, the distribution would have its left side missing as this part would not yet have been created. In terms of mapped size data, *ϕ*_obs_ would remain at ~0 until the roll-over was created, and 1/λ_obs_ would remain constant if the right hand tail were well-approximated by an exponential distribution.

$$f_{H_{\text{f}}}\left(h_{\text{f}}\right)=\{\begin{array}{cc} \frac{1}{k\left(t_{\text{f}}-c\right)}\text{,} & \text{for}\;H_{\text{i}}<h_{\text{f}}<H_{\text{i}}+k\left(t_{\text{f}}-c\right)\\ 0, & \text{elsewhere} \end{array}$$ (33)

If growth is exponential (Eq 4) [M5], the frequency of remnant forms is not exponential, but is inversely proportional to final height (Eq 34, Fig 10). This is verifiable intuitively since frequency in any height band is less the faster bedforms pass through it; specifically, bedform frequency is inversely proportional to their growth rate (i.e., 1/*kH*, Eq 4). In order to replicate an approximately log-normal distribution of *H*_f_ (e.g., Fig 1) with exponential growth, *t*_i_ must have a roughly Gaussian (i.e., normal) distribution [M5a]; a log-normal distribution is defined as that of a random variable whose logarithm is normally distributed, and Eq 4 can be written to give the logarithm of *H*_f_ as log(*H*_f_) = log(*H*_i_)+*k*(*t*_*i*_ − *c*) where everything on the right hand side is constant here except *t*_i_ which is a normal distribution. This can be verified by appropriate transformations of the random variables (e.g., Ch 5 of [93]). Giving *t*_i_ a normal distribution would, strictly, allow it to take values from −∞ to +∞, and so to apply to a period of bedform creation ranging between *c* and *t*_f_ only *ad hoc* Gaussians with small values outside this range could be employed. For mapped size data M5a predicts that 1/λ_obs_ would increase linearly with time along a trajectory set by the shape of the initial distribution, and *ϕ*_obs_ would remain at ~0 until the roll-over was created, then increase exponentially. Note that the SI model [M7] gives a mechanistic explanation for a Gaussian distribution of net growth durations rather than an *ad hoc* assumption of this in M5a.

$$f_{H_{\text{f}}}\left(h_{\text{f}}\right)=\{\begin{array}{cc} \frac{1}{h_{\text{f}}H_{\text{i}}e^{k\left(t_{\text{f}}-c\right)}}\text{,} & \text{for}\;H_{\text{i}}<h_{\text{f}}<H_{\text{i}}e^{k\left(t_{\text{f}}-c\right)}\\ 0, & \text{elsewhere} \end{array}$$ (34)

### A.4. Evaluation of models M1 to M5

With no randomness or variation [M1], the observations cannot be replicated. That is, no sharply spiked peaks are observed in size frequency distributions (Fig 1), casting serious doubt upon an entirely deterministic model. Thus, M1 is rejected. M2 and M3 are based on variations in initial bedform sizes, *H*_i_. Linear deterministic growth with uniformly distributed initial heights [M2] does not retain the small forms that are observed. Indeed, as explained above, there is no distribution of initial heights that can do so. Similarly, linearly unstable (i.e. exponential) deterministic growth [M3] does not intrinsically create an appropriate, exponentially tailed, size-frequency distribution. A progenitor landscape with log-normal *H*_i_ must be invoked to give the required log-normal *H*_f_ [M3a], but this *ad hoc* modification is somewhat questionable in a world where fractals (i.e., power-law distributions) dominate topography (e.g., [114]); even when suggesting that earlier progenitor log-normally sized forms may exist to be altered, the first set needs explaining. Thus, we provide the first observational constraint to indicate that something more appears to be needed than the ‘classic’ deterministic view of bedform growth and more obvious variants represented by models M1 to M3.

M4 and M5 are based on variations in growth initiation times, *t*_i_. Linear deterministic growth with a uniform distribution of initiation times [M4] does not match the size-frequency distribution. *Ad hoc* manipulation [M4a] is therefore needed. However, M4a invokes, without supporting evidence or analogy, a ‘reflected’ log-normal distribution of frequency that starts slowly, builds approximately exponentially to a peak, and dies rapidly before *t*_f_. Exponential growth, as illustrated by a uniform distribution of initiation times [M5], does not intrinsically lead to an approximately Gamma or log-Normal distribution of bedform sizes that is observed. A Gaussian distribution (i.e., *t*_i_ ∼ N(μ,σ)) would explain the observations [M5a], but it must be arbitrarily invoked. Thus, if bedforms are ‘born’ at different times (see [11,113]), it is demonstrated that a very specific pattern of ‘births’ is needed. Arguably, it would be preferable to have some process-related explanation for the required distribution of their initiation times.

## Appendix B: Parameter Estimation

Descriptions of the calculation of the exponent (λ) above a mode () and parameters of a gamma distribution (α_obs_, β_obs_) are given in Hillier et al. [19], which explicitly includes how counts from previously published size-frequency plots can be utilized. Fowler et al. [22] relays the standard formulae for a log-normal distribution where individual data are available (μ_obs_, σ_obs_), and how this may be done for digitisations of previously published size-frequency plots is given below. Worked examples for all parameters and all the data sets used in this paper are provided in EXCEL sheets as S1 File.

Maximum likelihood estimation of log-normal distribution parameters (μ, σ) using binned data, such as that digitised in Fig 1, adapts standard formulae used to calculate μ and σ for individual data in various areas of research (e.g., [22,115,116]). The mean, $\bar{x}$, and standard deviation, *s*_*x*_, of the sample are calculated to estimate μ and σ, respectively, using Eqs 35 and 36. *n* is the total number of data with counts, *c*_*j*_, of bins at *x*_*j*_.

$$\hat{\mu }=\bar{x}=\frac{1}{n}\sum c_{j}\text{ln(}x_{j}\text{)}$$ (35)

$$\hat{\sigma }=s_{x}=\sqrt{\frac{1}{n-1}\sum c_{j}{\left[ln\left(x_{j}\right)-\bar{ln\left(x\right)}\right]}^{2}}$$ (36)

## Supporting Information

S1 File Zip file, containing data and worked examples of parameter calculation in EXCEL sheets, and a README file explaining its contents. Also includes a summary table of notation used in the manuscript.
(ZIP)
