# Supplementary material for: Exploring Explanations of Subglacial Bedform Sizes Using Statistical Models
Source: PLoS One. 2016 Jul 26;11(7):e0159489. doi: 10.1371/journal.pone.0159489 (PMC4961447; doi:10.1371/journal.pone.0159489)
Supplement: S1 File — Also includes a summary table of notation used in the manuscript. (ZIP) [file pone.0159489.s001.zip › S1 File/Clark_2009_Brit_L_counts_digitized.xlsx.pdf]

Lenghts of British drumlins digitized from Fig.8 of Clark et al. [2009]

Centre of bin	Count
122	24.4826
172	309.713
222	1095.52
271	2139.78
319	2998.93
366	3532.12
417	3675.33
465	3381.98
516	3143.34
563	2671.88
613	2283.07
659	1867.48
709	1572.97
759	1278.45
809	1088.71
856	896.641
905	664.99
956	624.26
1002	562.574
1052	426.383
1100	357.713
1148	276.237
1196	283.235
1247	255.311
1295	187.804
1343	175.013
1393	152.908
1440	133.132
1489	108.699
1538	105.22
1589	81.9524
1637	72.6534
1684	58.6975
1734	47.0704
1780	58.7251
1831	49.4271
1879	30.8146
1931	30.8297
1977	44.8128
2026	25.0367
2073	30.8712
2125	25.0656
2175	9.94607
2221	9.95965
2272	8.8101
2318	8.82368
2369	8.83853
2420	8.85339
2516	8.88131
2859	10.1451

log-normal

μ	6.26
σ	0.46

Parameters calculated in Sheet 'Calculation - log-normal'. Note that these are very close to the values of 6.25 and 0.45 obtained from the frequencies used to create Fig. 8 in Clark et al. [2009].

Gamma

α	3.58
β (km^{-1})	6.10

Parameters calculated in Sheet 'Calculation - rest'. α is close to the value of 3.13 obtained by frequency used to create Fig. 8 in Clark et al. [2009]. β is close to the value obtained from the frequencies used to create Fig. 8 in Clark et al. [2009] (i.e. to 5.33).

Exponential tail

φ (m)	423.7
λ (km^{-1})	3.20

Mode is close to the value of 399 calculated for frequencies use to create the figure in Fig. 8Clark et al. (2009), and λ is also close (i.e. to 12.17)

The effect of digitizing a published figure, as compared to the frequencies originally used to create it, is <15%. This is easily insufficient to alter the conclusions of Hillier et al. [2016], and small enough to suggest that using parameters obtained by digitizing previous figures will be useful in compilations and comparisons.

Lenghts of British drumlins digitized from Fig.8 of Clark et al. [2009]

Parameter	Value		
n	36904.0899		
Mean of ln(xj)	6.26	i.e. μ	=SUM(E28:E77)/C6
Stdev of ln(xj)	0.46	i.e. σ	=SQRT((1/(C6-1))*SUM(G28:G77))

Parameters μ and σ of the log-normal distribution are calculated according to the equations below; see Appendix B of Hillier et al. [2016]. Columns D to G are used for stages of the calculation, with formulae used in the top row of the table explicitly shown.

$$\hat{\mu} = \bar{x} = \frac{1}{n}\sum c_j \ln(x_j)$$

$$\hat{\sigma} = s_x = \sqrt{\frac{1}{n-1}\sum c_j\left[ln(x_j) - \overline{ln\,(x)}\right]^2}$$

Centre of bin j i.e. (xj)	Count (Cj)	ln(xj) =LN(B28)	cj*ln(xj) =C28*D28	ln(xj) - mean of ln(x) =D28-C7	cj*{[ln(xj) - mean of ln(x)]^2} =C28*(F28^2)
122.328	24.4826	4.81	117.68	-1.46	51.90
172.446	309.713	5.15	1595.05	-1.11	383.43
222.48	1095.52	5.40	5921.11	-0.86	806.32
270.711	2139.78	5.60	11985.02	-0.66	936.89
318.973	2998.93	5.77	17289.15	-0.50	742.68
366.409	3532.12	5.90	20852.75	-0.36	455.22
417.431	3675.33	6.03	22177.38	-0.23	192.12
465.004	3381.98	6.14	20772.28	-0.12	49.27
516.089	3143.34	6.25	19634.18	-0.02	0.85
562.812	2671.88	6.33	16920.87	0.07	13.17
613.042	2283.07	6.42	14653.73	0.16	55.34
658.875	1867.48	6.49	12120.94	0.23	96.89
709.089	1572.97	6.56	10324.95	0.30	142.73
759.303	1278.45	6.63	8479.19	0.37	174.69
808.62	1088.71	6.70	7289.27	0.43	203.72
856.177	896.641	6.75	6054.55	0.49	215.04
904.62	664.99	6.81	4526.93	0.54	197.35
955.672	624.26	6.86	4283.93	0.60	224.48
1002.33	562.574	6.91	3887.43	0.65	235.74
1051.63	426.383	6.96	2966.81	0.70	206.16
1100.05	357.713	7.00	2505.10	0.74	196.07
1148.47	276.237	7.05	1946.42	0.78	169.55
1195.99	283.235	7.09	2007.21	0.82	192.30
1247.04	255.311	7.13	1819.99	0.87	191.37
1295.46	187.804	7.17	1345.92	0.90	153.43
1342.99	175.013	7.20	1260.56	0.94	154.61
1393.15	152.908	7.24	1106.95	0.98	145.83
1439.8	133.132	7.27	968.17	1.01	135.68
1489.09	108.699	7.31	794.15	1.04	118.29
1537.5	105.22	7.34	772.10	1.08	121.63
1588.55	81.9524	7.37	604.04	1.11	100.58
1636.95	72.6534	7.40	537.68	1.14	94.06
1684.48	58.6975	7.43	436.08	1.17	79.87
1733.77	47.0704	7.46	351.05	1.20	67.25
1779.53	58.7251	7.48	439.50	1.22	87.60
1831.46	49.4271	7.51	371.34	1.25	77.24
1878.98	30.8146	7.54	232.30	1.28	50.15
1930.91	30.8297	7.57	233.25	1.30	52.34
1976.67	44.8128	7.59	340.09	1.33	78.84
2025.96	25.0367	7.61	190.62	1.35	45.70
2073.48	30.8712	7.64	235.76	1.37	58.30
2125.41	25.0656	7.66	192.05	1.40	49.06
2174.7	9.94607	7.68	76.43	1.42	20.11
2221.34	9.95965	7.71	76.75	1.44	20.74
2271.51	8.8101	7.73	68.09	1.47	18.92
2318.15	8.82368	7.75	68.37	1.49	19.48
2369.2	8.83853	7.77	68.68	1.51	20.09
2420.24	8.85339	7.79	68.98	1.53	20.69
2516.17	8.88131	7.83	69.55	1.57	21.83
2858.52	10.1451	7.96	80.74	1.70	29.16

Lenghts of British drumlins digitized from Fig.8 of Clark et al. [2009]

Parameters α and β of the gamma distribution, and mode ϕ and gradient above it λ are calculated according to the equations below; see Hillier et al. [2013]. Columns D to I are used for stages of the calculation, with formulae used in the top row of the table explicitly shown. Similarly, flormulae used for the parameters are shown explicitly.

Parameter	Value	Formula
n	36904	=SUM(C33:C114)
Mean	587.71	=SUM(D33:D114)/C14
Standard Deviation	310.45	=SQRT((1/(C14-1))*SUM(E35:E84))
Alpha (α)	3.58	=(C15/C16)^2
Beta (β)	0.0061	=C15/(C16^2)
Mode (ϕ)	423.71	=(C17-1)/C18
Exponent (λ)	0.0032	=SUM(H35:H114)/SUM(I35:I84)

Sequence of calculation ↓

Alpha (α) $\hat{\alpha} = (\bar{x}/s_x)^2$

Mean $\bar{x} = \frac{1}{n}\sum c_j x_j$

β - Called lambda for Gamma (λ_g) in Hillier et al. [2013]

$\hat{\lambda}_g = \bar{x}/(s_x)^2$

Standard Deviation

$s_x = \sqrt{\frac{1}{n-1}\sum c_j (x_j - \bar{x})^2}$

Mode (φ)

$(\hat{\alpha} - 1)/\hat{\lambda}_g$

Gradient (λ)

$\hat{\lambda} = 1/\bar{k}$

k bar is the mean of values exceeding the mode. That is, it is only calculated for a value over the mode, and then only includes the amount by which it is over the mode.

Centre of bin j i.e. (xj)	Count (Cj)	xj*Cj =B35*C35	Cj*(xj - mean x)^2 =C35*((B35-C$15)^2	Above mode? =IF(B35-C$19 > 0, 1, 0)	Amount above mode =(B35-C$19)*F35	Cj sbove mode =F35*C35	xj*Cj above mode =G35*H35
122	24.4826	2994.90749	5302378.46	0	0	0	0.00
172	309.713	53408.768	53407383.45	0	0	0	0.00
222	1095.52	243731.29	146132084.67	0	0	0	0.00
271	2139.78	579261.984	215018650.66	0	0	0	0.00
319	2998.93	956577.699	216576287.94	0	0	0	0.00
366	3532.12	1294200.56	172977506.04	0	0	0	0.00
417	3675.33	1534196.68	106561955.93	0	0	0	0.00
465	3381.98	1572634.23	50919011.08	1	41	3382	139645.33
516	3143.34	1622243.2	16122533.03	1	92	3143	290369.17
563	2671.88	1503766.13	1655900.09	1	139	2672	371655.83
613	2283.07	1399617.8	1465439.87	1	189	2283	432251.36
659	1867.48	1230435.89	9458623.94	1	235	1867	439160.33
709	1572.97	1115375.72	23175572.44	1	285	1573	448887.89
759	1278.45	970730.92	37644288.64	1	336	1278	429035.03
809	1088.71	880352.68	53131925.72	1	385	1089	419052.10
856	896.641	767683.401	64626520.76	1	432	897	387764.95
905	664.99	601563.254	66787591.16	1	481	665	319798.35
956	624.26	596587.803	84523799.30	1	532	624	332080.72
1002	562.574	563884.797	96713446.75	1	579	563	325514.88
1052	426.383	448397.154	91768169.56	1	628	426	267733.13
1100	357.713	393502.186	93898073.40	1	676	358	241934.54
1148	276.237	317249.907	86864207.98	1	725	276	200204.70
1196	283.235	338746.228	104799344.75	1	772	283	218735.88
1247	255.311	318383.029	110988867.49	1	823	255	210204.44
1295	187.804	243292.57	94073764.84	1	872	188	163717.57
1343	175.013	235040.709	99836641.30	1	919	175	160885.43
1393	152.908	213023.78	99197345.27	1	969	153	148234.67
1440	133.132	191683.454	96662196.22	1	1016	133	135273.69
1489	108.699	161862.594	88317032.97	1	1065	109	115805.41
1538	105.22	161775.75	94919712.05	1	1114	105	117192.67
1589	81.9524	130185.485	82090663.28	1	1165	82	95461.19
1637	72.6534	118929.983	79984948.97	1	1213	73	88145.79
1684	58.6975	98874.7648	70607895.35	1	1261	59	74003.87
1734	47.0704	81609.2474	61825128.31	1	1310	47	61664.91
1780	58.7251	104503.077	83415630.62	1	1356	59	79620.49
1831	49.4271	90523.7566	76459869.86	1	1408	49	69580.85
1879	30.8146	57900.0171	51379847.57	1	1455	31	44843.47
1931	30.8297	59529.376	55622785.79	1	1507	31	46466.43
1977	44.8128	88580.1174	86453695.31	1	1553	45	69592.35
2026	25.0367	50723.3527	51790223.50	1	1602	25	40114.98
2073	30.8712	64010.8158	68148853.49	1	1650	31	50930.29
2125	25.0656	53274.6769	59268391.75	1	1702	25	42654.06
2175	9.94607	21629.7184	25049649.00	1	1751	10	17415.44
2221	9.95965	22123.7689	26579890.05	1	1798	10	17903.74
2272	8.8101	20012.2303	24978335.84	1	1848	9	16279.28
2318	8.82368	20454.6138	26421924.56	1	1894	9	16715.91
2369	8.83853	20940.2453	28051002.04	1	1945	9	17195.25
2420	8.85339	21427.3286	29731258.99	1	1997	9	17676.03
2516	8.88131	22346.8858	33029328.34	1	2092	9	18583.76
2859	10.1451	28999.9713	52314147.61	1	2435	10	24701.36
