# Supplementary material for: Exploring Explanations of Subglacial Bedform Sizes Using Statistical Models
Source: PLoS One. 2016 Jul 26;11(7):e0159489. doi: 10.1371/journal.pone.0159489 (PMC4961447; doi:10.1371/journal.pone.0159489)
Supplement: S1 File — Also includes a summary table of notation used in the manuscript. (ZIP) [file pone.0159489.s001.zip › S1 File/Clark_2009_Brit_L_counts_original.xlsx.pdf]

Lengths of British drumlins: the original frequencies input into Fig.8 of Clark et al. [2009]

Centre of bin	Count
25	0
75	1
125	23
175	239
225	896
275	1736
325	2294
375	2675
425	2617
475	2436
525	2170
575	1908
625	1548
675	1256
725	1082
775	852
825	693
875	553
925	428
975	407
1025	324
1075	277
1125	202
1175	177
1225	193
1275	150
1325	130
1375	122
1425	85
1475	80
1525	81
1575	60
1625	57
1675	48
1725	30
1775	35
1825	39
1875	25
1925	22
1975	31
2025	17
2075	27
2125	15
2175	10
2225	10
2275	10
2325	8
2375	8
2425	5
2475	4
2525	10
2575	4
2625	2
2675	6
2725	3
2775	2
2825	3
2875	9
2925	3
2975	3
3025	5
3075	2
3125	3
3175	2
3225	2
3275	2
3325	0
3375	1
3425	0
3475	0
3525	0
3575	2
3625	3
3675	0
3725	2
3775	0
3825	0
3875	0
3925	2
3975	0

**log-normal**	
μ	6.25
σ	0.47
**Gamma**	
α	3.13
β (km^{-1})	5.33
**Exponential tail**	
φ (m)	398.9
λ (km^{-1})	3.29

Parameters calculated in Sheet 'Calculation - log-normal'. Note that these are very close to the values of 6.26 and 0.46 obtained by digitizing Fig. 8 in Clark et al. [2009] as reported in Fig. 1 of Hillier et al. [2016]

Parameters calculated in Sheet 'Calculation - rest'. α is close to the value obtained by digitizing Fig. 8 in Clark et al. [2009] as reported in Fig. 1 of Hillier et al. [2016] (i.e., 3.58). β is also close (i.e. to 6.10)

Mode of is close to the 423 calculated for digitized values (e.g. Fig 2b of Hillier et al. [2013]), and λ is also close (i.e. to 13.49)

The effect of digitizing a published figure, as compared to the frequencies originally used to create it, is always <15%. This is easily insufficient to alter the conclusions of Hillier et al. [2016], and small enough to suggest that using parameters obtained by digitizing previous figures will be useful in compilations and comparisons.

More	11	11 drumlins have W > 4000, not included in plot in Clark et al. (2009)

Lengths of British drumlins: the original frequencies input into Fig.8 of Clark et al. [2009]

Parameter	Value		
n	26167		
Mean of ln(xj)	6.25	i.e. μ	=SUM(E28:E107)/C6
Stdev of ln(xj)	0.47	i.e. σ	=SQRT((1/(C6-1))*SUM(G28:G107))

Parameters μ and σ of the log-normal distribution are calculated according to the equations below; see Appendix B of Hillier et al. [2016]. Columns D to G are used for stages of the calculation, with formulae used in the top row of the table explicitly shown.

$$\hat{\mu} = \bar{x} = \frac{1}{n}\sum c_j \ln(x_j)$$

$$\hat{\sigma} = s_x = \sqrt{\frac{1}{n-1}\sum c_j\left[ln(x_j) - \overline{ln\,(x)}\right]^2}$$

Centre of bin j i.e. (xj)	Count (Cj)	ln(xj)<br>=LN(B28)	cj*ln(xj)<br>=C28*D28	ln(xj) - mean of ln(x)<br>=D28-C7	cj*{[ln(xj) - mean of ln(x)]^2}<br>=C28*(F28^2)
25	0	3.22	0.00	-3.04	0.00
75	1	4.32	4.32	-1.94	3.75
125	23	4.83	111.05	-1.43	46.75
175	239	5.16	1234.38	-1.09	283.52
225	896	5.42	4852.83	-0.84	628.99
275	1736	5.62	9750.71	-0.64	704.81
325	2294	5.78	13268.09	-0.47	507.01
375	2675	5.93	15854.53	-0.33	286.08
425	2617	6.05	15838.32	-0.20	106.64
475	2436	6.16	15013.83	-0.09	20.01
525	2170	6.26	13591.57	0.01	0.19
575	1908	6.35	12124.14	0.10	19.24
625	1548	6.44	9965.64	0.18	52.30
675	1256	6.51	8182.48	0.26	85.40
725	1082	6.59	7126.24	0.33	119.42
775	852	6.65	5668.24	0.40	135.58
825	693	6.72	4653.76	0.46	147.55
875	553	6.77	3746.15	0.52	149.69
925	428	6.83	2923.15	0.58	141.92
975	407	6.88	2801.15	0.63	160.76
1025	324	6.93	2246.11	0.68	149.16
1075	277	6.98	1933.48	0.73	146.05
1125	202	7.03	1419.16	0.77	120.26
1175	177	7.07	1251.22	0.82	117.59
1225	193	7.11	1372.36	0.86	141.66
1275	150	7.15	1072.61	0.90	120.62
1325	130	7.19	934.59	0.94	113.70
1375	122	7.23	881.60	0.97	115.33
1425	85	7.26	617.26	1.01	86.36
1475	80	7.30	583.71	1.04	86.94
1525	81	7.33	593.71	1.08	93.74
1575	60	7.36	441.72	1.11	73.67
1625	57	7.39	421.42	1.14	73.99
1675	48	7.42	356.33	1.17	65.66
1725	30	7.45	223.59	1.20	43.13
1775	35	7.48	261.85	1.23	52.75
1825	39	7.51	292.86	1.26	61.46
1875	25	7.54	188.41	1.28	41.11
1925	22	7.56	166.38	1.31	37.68
1975	31	7.59	235.24	1.33	55.20
2025	17	7.61	129.43	1.36	31.41
2075	27	7.64	206.22	1.38	51.70
2125	15	7.66	114.92	1.41	29.72
2175	10	7.68	76.85	1.43	20.47
2225	10	7.71	77.08	1.45	21.13
2275	10	7.73	77.30	1.48	21.78
2325	8	7.75	62.01	1.50	17.94
2375	8	7.77	62.18	1.52	18.45
2425	5	7.79	38.97	1.54	11.85
2475	4	7.81	31.26	1.56	9.73
2525	10	7.83	78.34	1.58	24.97
2575	4	7.85	31.41	1.60	10.24
2625	2	7.87	15.75	1.62	5.24
2675	6	7.89	47.35	1.64	16.09
2725	3	7.91	23.73	1.66	8.23
2775	2	7.93	15.86	1.67	5.61
2825	3	7.95	23.84	1.69	8.59
2875	9	7.96	71.67	1.71	26.31
2925	3	7.98	23.94	1.73	8.95
2975	3	8.00	23.99	1.74	9.13
3025	5	8.01	40.07	1.76	15.50
3075	2	8.03	16.06	1.78	6.32
3125	3	8.05	24.14	1.79	9.65
3175	2	8.06	16.13	1.81	6.55
3225	2	8.08	16.16	1.82	6.66
3275	2	8.09	16.19	1.84	6.77
3325	0	8.11	0.00	1.86	0.00
3375	1	8.12	8.12	1.87	3.50
3425	0	8.14	0.00	1.88	0.00
3475	0	8.15	0.00	1.90	0.00
3525	0	8.17	0.00	1.91	0.00
3575	2	8.18	16.36	1.93	7.43
3625	3	8.20	24.59	1.94	11.31
3675	0	8.21	0.00	1.96	0.00
3725	2	8.22	16.45	1.97	7.75
3775	0	8.24	0.00	1.98	0.00
3825	0	8.25	0.00	2.00	0.00
3875	0	8.26	0.00	2.01	0.00
3925	2	8.28	16.55	2.02	8.17
3975	0	8.29	0.00	2.03	0.00

Lengths of British drumlins: the original frequencies input into Fig.8 of Clark et al. [2009]

Parameters α and β of the gamma distribution, and mode ϕ and gradient above it λ are calculated according to the equations below; see Hillier et al. [2013]. Columns D to I are used for stages of the calculation, with formulae used in the top row of the table explicitly shown. Similarly, flormulae used for the parameters are shown explicitly.

Parameter	Value	Formula
n	26167	=SUM(C33:C114)
Mean	586.35	=SUM(D33:D114)/C14
Standard Deviation	331.54	=SQRT((1/(C14-1))*SUM(E35:E114))
Alpha (α)	3.13	=(C15/C16)^2
Beta (β)	0.0053	=C15/(C16^2)
Mode (ϕ)	398.89	=(C17-1)/C18
Exponent (λ)	0.0033	=SUM(H35:H114)/SUM(I35:I114)

Sequence of calculation ↓

Alpha (α) $$\hat{\alpha} = (\bar{x}/s_x)^2$$

Mean $$\bar{x} = \frac{1}{n}\sum c_j x_j$$

β - Called lambda for Gamma (λ_g) in Hillier et al. [2013]

$$\hat{\lambda}_g = \bar{x}/\left(s_x\right)^2$$

Gradient (λ) $$\hat{\lambda} = 1/\bar{k}$$

k bar is the mean of values exceeding the mode. That is, it is only calculated for a value over the mode, and then only includes the amount by which it is over the mode.

Standard Deviation

$$s_x = \sqrt{\frac{1}{n-1}\sum c_j\left(x_j - \bar{x}\right)^2}$$

Mode (φ) $$(\hat{\alpha} - 1)/\hat{\lambda}_g$$

Centre of bin j i.e. (xj)	Count (Cj)	xj*Cj<br>=B35*C35	Cj*(xj - mean x)^2<br>=C35*((B35-C$15)^2	Above mode?<br>=IF(B35-C$19 > 0, 1, 0)	Amount above mode<br>=(B35-C$19)*F35	Cj sbove mode<br>=F35*C35	xj*Cj above mode<br>=G35*H35
25	0	0	0.00	0	0	0	0.00
75	1	75	261477.05	0	0	0	0.00
125	23	2875	4895371.06	0	0	0	0.00
175	239	41825	40440567.06	0	0	0	0.00
225	896	201600	116993020.24	0	0	0	0.00
275	1736	477400	168283918.24	0	0	0	0.00
325	2294	745550	156686886.13	0	0	0	0.00
375	2675	1003125	119487136.22	0	0	0	0.00
425	2617	1112225	68129046.65	1	26	2617	68338.42
475	2436	1157100	30202589.34	1	76	2436	185411.92
525	2170	1139250	8167032.36	1	126	2170	273665.79
575	1908	1097100	245718.11	1	176	1908	336024.11
625	1548	967500	2312645.08	1	226	1548	350023.34
675	1256	847800	9871067.86	1	276	1256	346798.26
725	1082	784450	20800697.09	1	326	1082	352854.55
775	852	660300	30322235.12	1	376	852	320448.50
825	693	571725	39469573.51	1	426	693	295296.49
875	553	483875	46075863.35	1	476	553	263290.64
925	428	395900	49085179.54	1	526	428	225176.48
975	407	396825	61477420.25	1	576	407	234478.10
1025	324	332100	62342572.20	1	626	324	202860.70
1075	277	297775	66142204.04	1	676	277	187283.37
1125	202	227250	58609430.12	1	726	202	146674.88
1175	177	207975	61332423.53	1	776	177	137372.05
1225	193	236425	78720075.93	1	826	193	159439.86
1275	150	191250	71136182.21	1	876	150	131416.99
1325	130	172250	70928830.50	1	926	130	120394.72
1375	122	167750	75880530.58	1	976	122	119085.82
1425	85	121125	59783622.55	1	1026	85	87219.63
1475	80	118000	63176152.76	1	1076	80	86089.06
1525	81	123525	71366433.74	1	1126	81	91215.17
1575	60	94500	58645935.41	1	1176	60	70566.80
1625	57	92625	61491453.54	1	1226	57	69888.46
1675	48	80400	56887805.01	1	1276	48	61253.44
1725	30	51750	38895833.34	1	1326	30	39783.40
1775	35	62125	49451253.31	1	1376	35	48163.96
1825	39	71175	59836066.89	1	1426	39	55618.42
1875	25	46875	41515582.48	1	1476	25	36902.83
1925	22	42350	39423746.40	1	1526	22	33574.49
1975	31	61225	59778963.04	1	1576	31	48859.51
2025	17	34425	35185219.94	1	1626	17	27643.93
2075	27	56025	59834267.83	1	1676	27	45255.06
2125	15	31875	35511737.51	1	1726	15	25891.70
2175	10	21750	25238143.41	1	1776	10	17761.13
2225	10	22250	26851795.15	1	1826	10	18261.13
2275	10	22750	28515446.89	1	1876	10	18761.13
2325	8	18600	24183278.90	1	1926	8	15408.91
2375	8	19000	25594200.29	1	1976	8	15808.91
2425	5	12125	16903201.05	1	2026	5	10130.57
2475	4	9900	14268021.53	1	2076	4	8304.45
2525	10	25250	37583705.57	1	2126	10	21261.13
2575	4	10300	15818942.92	1	2176	4	8704.45
2625	2	5250	8312201.81	1	2226	2	4452.23
2675	6	16050	26174796.47	1	2276	6	13656.68
2725	3	8175	13721493.76	1	2326	3	6978.34
2775	2	5550	9580392.85	1	2376	2	4752.23
2825	3	8475	15034684.80	1	2426	3	7278.34
2875	9	25875	47141340.96	1	2476	9	22285.02
2925	3	8775	16407875.84	1	2526	3	7578.34
2975	3	8925	17116971.36	1	2576	3	7728.34
3025	5	15125	29735111.47	1	2626	5	13130.57
3075	2	6150	12386774.94	1	2676	2	5352.23
3125	3	9375	19334257.92	1	2726	3	8178.34
3175	2	6350	13402235.63	1	2776	2	5552.23
3225	2	6450	13924965.98	1	2826	2	5652.23
3275	2	6550	14457696.32	1	2876	2	5752.23
3325	0	0	0.00	1	2926	0	0.00
3375	1	3375	7776578.51	1	2976	1	2976.11
3425	0	0	0.00	1	3026	0	0.00
3475	0	0	0.00	1	3076	0	0.00
3525	0	0	0.00	1	3126	0	0.00
3575	2	7150	17864078.41	1	3176	2	6352.23
3625	3	10875	27700213.13	1	3226	3	9678.34
3675	0	0	0.00	1	3276	0	0.00
3725	2	7450	19702269.45	1	3326	2	6652.23
3775	0	0	0.00	1	3376	0	0.00
3825	0	0	0.00	1	3426	0	0.00
3875	0	0	0.00	1	3476	0	0.00
3925	2	7850	22293190.84	1	3526	2	7052.23
3975	0	0	0.00	1	3576	0	0.00
