# Supplementary material for: Exploring Explanations of Subglacial Bedform Sizes Using Statistical Models
Source: PLoS One. 2016 Jul 26;11(7):e0159489. doi: 10.1371/journal.pone.0159489 (PMC4961447; doi:10.1371/journal.pone.0159489)
Supplement: S1 File — Also includes a summary table of notation used in the manuscript. (ZIP) [file pone.0159489.s001.zip › S1 File/Clark_2009_Brit_W_counts_digitized.xlsx.pdf]

Widths of British drumlins digitized from Fig.8 of Clark et al. [2009]

Centre of bin	Count
61	18.06018
71	81.28343
81	167.02087
91	335.30484
101	691.19672
110	1108.195
120	1398.691
130	1769.5922
140	2017.2072
150	2185.4919
159	2294.8135
169	2214.3838
179	2349.4358
189	2146.793
199	2103.8848
208	1879.8021
218	1766.1392
227	1565.6429
237	1368.3607
247	1202.1677
257	988.80566
267	945.89746
276	818.29865
286	744.30194
295	656.36932
305	565.21991
315	474.06985
325	427.94489
335	356.09222
345	308.89587
354	272.42126
364	184.48732
374	209.11841
384	143.69667
393	141.52672
403	128.63701
413	97.52061
422	81.41478
432	87.82182
442	63.13767
451	75.9763
462	62.01189
471	43.76264
481	43.73544
491	39.42207
501	34.03532
510	39.36966
520	26.47863
530	25.38071
540	33.92985
549	25.32831
560	13.50732
570	25.27324
580	14.52629
590	12.35568
600	20.90481
610	20.87761
620	13.34546
630	13.31892
641	6.85748
673	12.13145
703	5.61959
723	6.6379
733	4.46662

log-normal

μ	5.27
σ	0.36

Parameters calculated in Sheet 'Calculation - log-normal'. Note that these are very close to the values of 5.28 and 0.37 obtained from the frequencies used to create Fig. 8 in Clark et al. [2009].

Gamma

α	6.68
β (km^{-1})	32.14

Parameters calculated in Sheet 'Calculation - rest'. Note that these are close to the values of 6.26 and 29.79 obtained from the frequencies used to create Fig. 8 in Clark et al. [2009].

Exponential tail

φ (m)	176.7
λ (km^{-1})	13.49

Mode of 177 m matches that calculated for digitized values (e.g. Fig 2b of Hillier et al. [2013]), and λ is close to the value obtained from the frequencies used to create Fig. 8 in Clark et al. [2009 (i.e. to 12.17)

The effect of digitizing a published figure, as compared to the frequencies originally used to create it, is <~10%. This is easily insufficient to alter the conclusions of Hillier et al. [2016], and small enough to suggest that using parameters obtained by digitizing previous figures will be useful in compilations and comparisons.

Widths of British drumlins digitized from Fig.8 of Clark et al. [2009]

Parameter	Value		
**n**	36978.398		
**Mean of ln(xj)**	5.27	i.e. μ	=SUM(E28:E91)/C6
**Stdev of ln(xj)**	0.36	i.e. σ	=SQRT((1/(C6-1))*SUM(G28:G91))

Parameters μ and σ of the log-normal distribution are calculated according to the equations below; see Appendix B of Hillier et al. [2016]. Columns D to G are used for stages of the calculation, with formulae used in the top row of the table explicitly shown.

$$\hat{\mu} = \bar{x} = \frac{1}{n} \sum c_j \ln(x_j)$$

$$\hat{\sigma} = s_x = \sqrt{\frac{1}{n-1} \sum c_j \left[ln(x_j) - \overline{ln\,(x)} \right]^2}$$

Centre of bin j i.e. (xj)	Count (Cj)	ln(xj) =LN(B28)	cj*ln(xj) =C28*D28	ln(xj) - mean of ln(x) =D28-C7	cj*{[ln(xj) - mean of ln(x)]^2} =C28*(F28^2)
61.14135	18.06018	4.11	74.28	-1.16	24.21
71.2989	81.28343	4.27	346.83	-1.00	81.96
80.96461	167.02087	4.39	733.89	-0.88	128.47
90.88965	335.30484	4.51	1512.11	-0.76	194.38
100.59396	691.19672	4.61	3187.17	-0.66	301.04
110.30701	1108.19495	4.70	5212.14	-0.57	357.24
120.49702	1398.69104	4.79	6702.00	-0.48	321.47
129.95596	1769.59216	4.87	8612.95	-0.40	288.60
139.89233	2017.20715	4.94	9966.76	-0.33	219.89
149.56984	2185.49194	5.01	10944.43	-0.26	151.48
159.48645	2294.81348	5.07	11639.20	-0.20	90.95
169.37595	2214.38379	5.13	11364.49	-0.14	42.73
178.80118	2349.43579	5.19	12184.82	-0.08	16.88
188.92075	2146.79297	5.24	11252.05	-0.03	1.90
198.8156	2103.88477	5.29	11134.55	0.02	0.96
208.43706	1879.80212	5.34	10037.46	0.07	8.85
218.32181	1766.13916	5.39	9512.37	0.11	23.33
227.45157	1565.64294	5.43	8496.65	0.16	38.05
237.32437	1368.36072	5.47	7484.15	0.20	53.86
247.20161	1202.16772	5.51	6624.19	0.24	68.76
256.82458	988.80566	5.55	5486.28	0.28	76.06
266.71945	945.89746	5.59	5283.97	0.32	93.95
276.35471	818.29865	5.62	4600.22	0.35	100.61
285.99759	744.30194	5.66	4209.76	0.38	110.29
295.39096	656.36932	5.69	3733.63	0.42	114.28
305.0314	565.21991	5.72	3233.29	0.45	114.14
314.91937	474.06985	5.75	2727.00	0.48	109.81
325.06131	427.94489	5.78	2475.24	0.51	112.61
334.7045	356.09222	5.81	2070.05	0.54	104.69
344.59875	308.89587	5.84	1804.69	0.57	100.83
353.99948	272.42126	5.87	1598.92	0.60	97.50
363.88791	184.48732	5.90	1087.89	0.63	72.25
373.54489	209.11841	5.92	1238.62	0.65	88.90
383.68405	143.69667	5.95	854.97	0.68	66.21
393.33722	141.52672	5.97	845.58	0.70	70.07
402.7413	128.63701	6.00	771.60	0.73	68.04
412.88538	97.52061	6.02	587.38	0.75	55.17
422.289	81.41478	6.05	492.21	0.77	48.86
431.69586	87.82182	6.07	532.88	0.80	55.74
441.84085	63.13767	6.09	384.57	0.82	42.44
451.49615	75.9763	6.11	464.41	0.84	53.80
461.8902	62.01189	6.14	380.46	0.86	46.32
471.04602	43.76264	6.15	269.36	0.88	34.19
481.19452	43.73544	6.18	270.12	0.91	35.84
490.59985	39.42207	6.20	244.24	0.92	33.70
500.50006	34.03532	6.22	211.55	0.94	30.37
510.1543	39.36966	6.23	245.46	0.96	36.56
520.05347	26.47863	6.25	165.60	0.98	25.58
529.70673	25.38071	6.27	159.20	1.00	25.45
539.85651	33.92985	6.29	213.46	1.02	35.32
549.26117	25.32831	6.31	159.79	1.04	27.27
559.90308	13.50732	6.33	85.47	1.06	15.08
569.80573	25.27324	6.35	160.37	1.07	29.17
579.7052	14.52629	6.36	92.42	1.09	17.31
589.6059	12.35568	6.38	78.82	1.11	15.18
599.75562	20.90481	6.40	133.72	1.13	26.48
609.90411	20.87761	6.41	133.89	1.14	27.24
620.29907	13.34546	6.43	85.81	1.16	17.93
630.20007	13.31892	6.45	85.85	1.17	18.39
641.09027	6.85748	6.46	44.32	1.19	9.75
673.26923	12.13145	6.51	79.00	1.24	18.69
702.97125	5.61959	6.56	36.84	1.28	9.27
723.02087	6.6379	6.58	43.70	1.31	11.43
733.16913	4.46662	6.60	29.47	1.33	7.86

Widths of British drumlins digitized from Fig.8 of Clark et al. [2009]

Parameters α and β of the gamma distribution, and mode ϕ and gradient above it λ are calculated according to the equations below; see Hillier et al. [2013]. Columns D to I are used for stages of the calculation, with formulae used in the top row of the table explicitly shown. Similarly, flormulae used for the parameters are shown explicitly.

Parameter	Value	Formula
n	36978	=SUM(C33:C114)
Mean	207.84	=SUM(D33:D114)/C14
Standard Deviation	80.41	=SQRT((1/(C14-1))*SUM(E35:E98))
Alpha (α)	6.68	=(C15/C16)^2
Beta (β)	0.0321	=C15/(C16^2)
Mode (ϕ)	176.73	=(C17-1)/C18
Exponent (λ)	0.0135	=SUM(H35:H114)/SUM(I35:I98)

Sequence of calculation ↓

Alpha (α) $\hat{\alpha} = (\bar{x}/s_x)^2$

Mean $\bar{x} = \frac{1}{n}\sum c_j x_j$

β - Called lambda for Gamma (λ_g) in Hillier et al. [2013]

$\hat{\lambda}_g = \bar{x}/(s_x)^2$

Gradient (λ) $\hat{\lambda} = 1/\bar{k}$

k bar is the mean of values exceeding the mode. That is, it is only calculated for a value over the mode, and then only includes the amount by which it is over the mode.

Standard Deviation

$s_x = \sqrt{\frac{1}{n-1}\sum c_j (x_j - \bar{x})^2}$

Mode (φ)

$(\hat{\alpha} - 1)/\hat{\lambda}_g$

Centre of bin j i.e. (xj)	Count (Cj)	xj*Cj<br>=B35*C35	Cj*(xj - mean x)^2<br>=C35*((B35-C$15)^2	Above mode?<br>=IF(B35-C$19 > 0, 1, 0)	Amount above mode<br>=(B35-C$19)*F35	Cj sbove mode<br>=F35*C35	xj*Cj above mode<br>=G35*H35
61	18.06018	1104.22379	388682.77	0	0	0	0.00
71	81.28343	5795.41915	1515484.01	0	0	0	0.00
81	167.02087	13522.7796	2688746.03	0	0	0	0.00
91	335.30484	30475.7396	4586371.12	0	0	0	0.00
101	691.19672	69530.2152	7950471.77	0	0	0	0.00
110	1108.19495	122241.671	10542674.61	0	0	0	0.00
120	1398.69104	168538.102	10671194.72	0	0	0	0.00
130	1769.59216	229969.048	10735187.87	0	0	0	0.00
140	2017.20715	282191.808	9314186.66	0	0	0	0.00
150	2185.49194	326883.68	7421547.20	0	0	0	0.00
159	2294.81348	365991.655	5366211.40	0	0	0	0.00
169	2214.38379	375063.358	3276747.20	0	0	0	0.00
179	2349.43579	420081.892	1981652.52	1	2	2349	4860.93
189	2146.79297	405573.738	768706.72	1	12	2147	26166.29
199	2103.88477	418285.113	171474.54	1	22	2104	46460.92
208	1879.80212	391820.427	662.18	1	32	1880	59598.86
218	1766.13916	385586.698	193911.59	1	42	1766	73453.04
227	1565.64294	356107.945	601950.08	1	51	1566	79408.42
237	1368.36072	324745.346	1189268.45	1	61	1368	82911.94
247	1202.16772	297177.796	1862226.77	1	70	1202	84716.05
257	988.80566	253949.598	2372285.09	1	80	989	79195.80
267	945.89746	252289.25	3278832.78	1	90	946	85118.71
276	818.29865	226140.686	3840913.75	1	100	818	81520.96
286	744.30194	212868.561	4546237.13	1	109	744	81326.44
295	656.36932	193885.564	5030775.52	1	119	656	77883.97
305	565.21991	172409.82	5338773.01	1	128	565	72517.26
315	474.06985	149293.778	5435321.07	1	138	474	65510.37
325	427.94489	139108.327	5879964.80	1	148	428	63476.68
335	356.09222	119185.668	5730842.14	1	158	356	56252.71
345	308.89587	106445.131	5776966.36	1	168	309	51853.28
354	272.42126	96436.9844	5819342.46	1	177	272	48291.38
364	184.48732	67132.7053	4492237.50	1	187	184	34527.85
374	209.11841	78115.1135	5741750.82	1	197	209	41157.16
384	143.69667	55134.1203	4443084.31	1	207	144	29738.29
393	141.52672	55667.7266	4869637.78	1	217	142	30655.40
403	128.63701	51807.4366	4886294.24	1	226	129	29073.13
413	97.52061	40264.8341	4099976.56	1	236	98	23029.80
422	81.41478	34380.566	3744009.57	1	246	81	19991.95
432	87.82182	37912.3161	4400739.06	1	255	88	22391.37
442	63.13767	27896.8018	3457086.66	1	265	63	16738.34
451	75.9763	34303.0069	4510454.04	1	275	76	20875.55
462	62.01189	28642.6843	4002228.99	1	285	62	17683.19
471	43.76264	20614.2174	3031680.67	1	294	44	12879.95
481	43.73544	21045.2541	3267945.15	1	304	44	13315.79
491	39.42207	19340.4616	3151838.99	1	314	39	12373.31
501	34.03532	17034.6797	2915051.52	1	324	34	11019.54
510	39.36966	20084.6013	3598063.83	1	333	39	13126.71
520	26.47863	13770.3034	2581005.47	1	343	26	9090.68
530	25.38071	13444.3329	2629337.76	1	353	25	8958.74
540	33.92985	18317.2504	3740175.91	1	363	34	12320.75
549	25.32831	13911.8572	2952419.67	1	373	25	9435.53
560	13.50732	7562.79007	1674177.16	1	383	14	5175.61
570	25.27324	14400.837	3311214.59	1	393	25	9934.24
580	14.52629	8420.96585	2008711.23	1	403	15	5853.70
590	12.35568	7284.98183	1800747.65	1	413	12	5101.34
600	20.90481	12537.7773	3210875.87	1	423	21	8843.22
610	20.87761	12733.3401	3374922.00	1	433	21	9043.59
620	13.34546	8278.17643	2270323.80	1	444	13	5919.60
630	13.31892	8393.58432	2375896.01	1	453	13	6039.70
641	6.85748	4396.2637	1287167.69	1	464	7	3184.33
673	12.13145	8167.732	2627927.67	1	497	12	6023.71
703	5.61959	3950.41021	1377650.61	1	526	6	2957.25
723	6.6379	4799.34023	1761749.62	1	546	7	3626.21
733	4.46662	3274.7879	1232639.59	1	556	4	2485.39
