# Supplementary material for: Exploring Explanations of Subglacial Bedform Sizes Using Statistical Models
Source: PLoS One. 2016 Jul 26;11(7):e0159489. doi: 10.1371/journal.pone.0159489 (PMC4961447; doi:10.1371/journal.pone.0159489)
Supplement: S1 File — Also includes a summary table of notation used in the manuscript. (ZIP) [file pone.0159489.s001.zip › S1 File/Clark_2009_Brit_W_counts_original.xlsx.pdf]

Widths of British drumlins: the original frequencies input into Fig.8 of Clark et al. [2009]

Centre of bin	Count
5	0
15	0
25	0
35	1
45	1
55	6
65	28
75	90
85	189
95	353
105	648
115	864
125	1133
135	1320
145	1457
155	1605
165	1569
175	1641
185	1534
195	1546
205	1388
215	1332
225	1157
235	986
245	952
255	756
265	709
275	631
285	568
295	508
305	430
315	380
325	336
335	270
345	220
355	215
365	156
375	141
385	119
395	118
405	94
415	71
425	62
435	57
445	43
455	51
465	36
475	32
485	28
495	30
505	21
515	26
525	18
535	18
545	21
555	20
565	19
575	16
585	12
595	10
605	12
615	14
625	12
635	9
645	7
655	7
665	4
675	11
685	7
695	6
705	3
715	2
725	4
735	1
745	4
755	0
765	6
775	2
785	0
795	1

**log-normal**		
μ	5.28	Parameters calculated in Sheet 'Calculation - log-normal'. Note that these are very close to the values of 5.27 and 0.36 obtained by digitizing Fig. 8 in Clark et al. [2009] as reported in Fig. 1 of Hillier et al. [2016]
σ	0.37	
**Gamma**		
α	6.26	Parameters calculated in Sheet 'Calculation - rest'. α is close to the value of 6.68 obtained by digitizing Fig. 8 in Clark et al. [2009] as reported in Fig. 1 of Hillier et al. [2016] (i.e., 6.68). β is also close (i.e. to 32.14)
β (km^{-1})	29.79	
**Exponential tail**		
φ (m)	176.673	Mode of 177 m matches that calculated for digitized values (e.g. Fig 2b of Hillier et al. [2013]), and λ is also close (i.e. to 13.49)
λ (km^{-1})	12.17	

The effect of digitizing a published figure, as compared to the frequencies originally used to create it, is <~10%. This is easily insufficient to alter the conclusions of Hillier et al. [2016], and small enough to suggest that using parameters obtained by digitizing previous figures will be useful in compilations and comparisons.

More 24 — 24 drumlins have W > 800, not included in plot in Clark et al. (2009)

Widths of British drumlins digitised from Fig.8 of Clark et al. [2009]

Parameter	Value		
n	26154		
Mean of ln(xj)	5.28	i.e. μ	=SUM(E28:E107)/C6
Stdev of ln(xj)	0.37	i.e. σ	=SQRT((1/(C6-1))*SUM(G28:G107))

Parameters μ and σ of the log-normal distribution are calculated according to the equations below; see Appendix B of Hillier et al. [2016]. Columns D to G are used for stages of the calculation, with formulae used in the top row of the table explicitly shown.

$$\hat{\mu} = \bar{x} = \frac{1}{n}\sum c_j \ln(x_j)$$

$$\hat{\sigma} = s_x = \sqrt{\frac{1}{n-1}\sum c_j\left[ln(x_j) - \overline{ln\,(x)}\right]^2}$$

Centre of bin j i.e. (xj)	Count (Cj)	ln(xj) =LN(B28)	cj*ln(xj) =C28*D28	ln(xj) - mean of ln(x) =D28-C7	cj*{[ln(xj) - mean of ln(x)]^2} =C28*(F28^2)
5	0	1.61	0.00	-3.67	0.00
15	0	2.71	0.00	-2.57	0.00
25	0	3.22	0.00	-2.06	0.00
35	1	3.56	3.56	-1.72	2.97
45	1	3.81	3.81	-1.47	2.17
55	6	4.01	24.04	-1.27	9.70
65	28	4.17	116.88	-1.10	34.16
75	90	4.32	388.57	-0.96	83.20
85	189	4.44	839.66	-0.84	132.18
95	353	4.55	1607.52	-0.73	185.58
105	648	4.65	3015.77	-0.62	253.11
115	864	4.74	4099.62	-0.53	246.39
125	1133	4.83	5470.48	-0.45	230.08
135	1320	4.91	6474.96	-0.37	184.31
145	1457	4.98	7251.10	-0.30	133.07
155	1605	5.04	8094.70	-0.24	89.03
165	1569	5.11	8011.23	-0.17	46.96
175	1641	5.16	8475.41	-0.11	21.39
185	1534	5.22	8008.03	-0.06	5.27
195	1546	5.27	8152.06	-0.01	0.05
205	1388	5.32	7388.34	0.04	2.70
215	1332	5.37	7153.69	0.09	11.20
225	1157	5.42	6266.43	0.14	21.76
235	986	5.46	5383.15	0.18	32.17
245	952	5.50	5237.20	0.22	47.05
255	756	5.54	4189.20	0.26	52.02
265	709	5.58	3956.03	0.30	64.14
275	631	5.62	3544.18	0.34	72.01
285	568	5.65	3210.61	0.37	79.26
295	508	5.69	2888.98	0.41	84.58
305	430	5.72	2459.73	0.44	83.77
315	380	5.75	2185.98	0.47	85.24
325	336	5.78	1943.37	0.50	85.65
335	270	5.81	1569.82	0.54	77.33
345	220	5.84	1285.58	0.56	70.13
355	215	5.87	1262.51	0.59	75.65
365	156	5.90	920.38	0.62	60.15
375	141	5.93	835.70	0.65	59.20
385	119	5.95	708.44	0.67	54.11
395	118	5.98	705.51	0.70	57.81
405	94	6.00	564.37	0.72	49.40
415	71	6.03	428.01	0.75	39.87
425	62	6.05	375.23	0.77	37.06
435	57	6.08	346.29	0.80	36.15
445	43	6.10	262.22	0.82	28.85
455	51	6.12	312.14	0.84	36.10
465	36	6.14	221.11	0.86	26.82
475	32	6.16	197.23	0.88	25.03
485	28	6.18	173.16	0.91	22.94
495	30	6.20	186.14	0.93	25.70
505	21	6.22	130.72	0.95	18.78
515	26	6.24	162.35	0.97	24.22
525	18	6.26	112.74	0.98	17.44
535	18	6.28	113.08	1.00	18.12
545	21	6.30	132.32	1.02	21.93
555	20	6.32	126.38	1.04	21.63
565	19	6.34	120.40	1.06	21.26
575	16	6.35	101.67	1.08	18.50
585	12	6.37	76.46	1.09	14.33
595	10	6.39	63.89	1.11	12.31
605	12	6.41	76.86	1.13	15.22
615	14	6.42	89.90	1.14	18.28
625	12	6.44	77.25	1.16	16.11
635	9	6.45	58.08	1.17	12.42
645	7	6.47	45.28	1.19	9.92
655	7	6.48	45.39	1.21	10.18
665	4	6.50	26.00	1.22	5.96
675	11	6.51	71.66	1.24	16.80
685	7	6.53	45.71	1.25	10.95
695	6	6.54	39.26	1.26	9.60
705	3	6.56	19.67	1.28	4.91
715	2	6.57	13.14	1.29	3.35
725	4	6.59	26.34	1.31	6.84
735	1	6.60	6.60	1.32	1.74
745	4	6.61	26.45	1.33	7.12
755	0	6.63	0.00	1.35	0.00
765	6	6.64	39.84	1.36	11.11
775	2	6.65	13.31	1.37	3.78
785	0	6.67	0.00	1.39	0.00
795	1	6.68	6.68	1.40	1.96

Widths of British drumlins digitised from Fig.8 of Clark et al. [2009]

Parameters α and β of the gamma distribution, and mode ϕ and gradient above it λ are calculated according to the equations below; see Hillier et al. [2013]. Columns D to I are used for stages of the calculation, with formulae used in the top row of the table explicitly shown. Similarly, flormulae used for the parameters are shown explicitly.

Parameter	Value	Formula
n	26154	=SUM(C33:C114)
Mean	210.24	=SUM(D33:D114)/C14
Standard Deviation	84.00	=SQRT((1/(C14-1))*SUM(E35:E114))
Alpha (α)	6.26	=(C15/C16)^2
Beta (β)	0.0298	=C15/(C16^2)
Mode (ϕ)	176.67	=(C17-1)/C18
Exponent (λ)	0.0122	=SUM(H35:H114)/SUM(I35:I114)

Sequence of calculation ↓

Alpha (α) $\hat{\alpha} = (\bar{x}/s_x)^2$

Mean $\bar{x} = \frac{1}{n}\sum c_j x_j$

β - Called lambda for Gamma (λ_g) in Hillier et al. [2013]

$\hat{\lambda}_g = \bar{x}/(s_x)^2$

Standard Deviation

$s_x = \sqrt{\frac{1}{n-1}\sum c_j (x_j - \bar{x})^2}$

Mode (φ)

$(\hat{\alpha} - 1)/\hat{\lambda}_g$

Gradient (λ)

$\hat{\lambda} = 1/\bar{k}$

k bar is the mean of values exceeding the mode. That is, it is only calculated for a value over the mode, and then only includes the amount by which it is over the mode.

Centre of bin j i.e. (xj)	Count (Cj)	xj*Cj<br>=B35*C35	Cj*(xj - mean x)^2<br>=C35*((B35-C$15)^2	Above mode?<br>=IF(B35-C$19 > 0, 1, 0)	Amount above mode<br>=(B35-C$19)*F35	Cj sbove mode<br>=F35*C35	xj*Cj above mode<br>=G35*H35
5	0	0	0.00	0	0	0	0.00
15	0	0	0.00	0	0	0	0.00
25	0	0	0.00	0	0	0	0.00
35	1	35	30707.49	0	0	0	0.00
45	1	45	27302.78	0	0	0	0.00
55	6	330	144588.41	0	0	0	0.00
65	28	1820	590614.04	0	0	0	0.00
75	90	6750	1645978.32	0	0	0	0.00
85	189	16065	2964264.18	0	0	0	0.00
95	353	33535	4687567.10	0	0	0	0.00
105	648	68040	7176286.60	0	0	0	0.00
115	864	99360	7836312.21	0	0	0	0.00
125	1133	141625	8231352.90	0	0	0	0.00
135	1320	178200	7471707.77	0	0	0	0.00
145	1457	211265	6200517.19	0	0	0	0.00
155	1605	248775	4896796.51	0	0	0	0.00
165	1569	258885	3210570.95	0	0	0	0.00
175	1641	287175	2037371.14	0	0	0	0.00
185	1534	283790	976900.10	1	8	1534	12773.87
195	1546	301470	358859.55	1	18	1546	28333.79
205	1388	284540	38046.13	1	28	1388	39318.10
215	1332	286380	30236.66	1	38	1332	51051.78
225	1157	260325	252214.00	1	48	1157	55914.53
235	986	231710	604693.17	1	58	986	57510.58
245	952	233240	1150557.22	1	68	952	65047.46
255	756	192780	1514916.61	1	78	756	59215.34
265	709	187885	2126395.50	1	88	709	62623.96
275	631	173525	2646689.64	1	98	631	62044.44
285	568	161880	3174964.52	1	108	568	61529.83
295	508	149860	3649987.98	1	118	508	60110.20
305	430	131150	3861531.21	1	128	430	55180.68
315	380	119700	4170725.94	1	138	380	52564.32
325	336	109200	4425417.03	1	148	336	49837.93
335	270	90450	4202866.84	1	158	270	42748.33
345	220	75900	3995521.84	1	168	220	37031.98
355	215	76325	4505701.75	1	178	215	38340.34
365	156	56940	3736518.52	1	188	156	29379.04
375	141	52875	3827773.70	1	198	141	27964.13
385	119	45815	3634571.86	1	208	119	24790.93
395	118	46610	4028273.39	1	218	118	25762.61
405	94	38070	3565720.76	1	228	94	21462.75
415	71	29465	2976922.72	1	238	71	16921.23
425	62	26350	2859674.26	1	248	62	15396.28
435	57	24795	2879586.87	1	258	57	14724.65
445	43	19135	2369917.36	1	268	43	11538.07
455	51	23205	3055391.98	1	278	51	14194.69
465	36	16740	2336577.70	1	288	36	10379.78
475	32	15200	2243207.22	1	298	32	9546.47
485	28	13580	2113874.42	1	308	28	8633.16
495	30	14850	2432724.13	1	318	30	9549.81
505	21	10605	1824607.97	1	328	21	6894.87
515	26	13390	2414915.97	1	338	26	8796.51
525	18	9450	1783380.11	1	348	18	6269.89
535	18	9630	1898495.32	1	358	18	6449.89
545	21	11445	2353412.29	1	368	21	7734.87
555	20	11100	2377250.82	1	378	20	7566.54
565	19	10735	2391298.78	1	388	19	7378.22
575	16	9200	2128849.92	1	398	16	6373.23
585	12	7020	1685380.91	1	408	12	4899.93
595	10	5950	1480436.99	1	418	10	4183.27
605	12	7260	1870067.86	1	428	12	5139.93
615	14	8610	2293679.89	1	438	14	6136.58
625	12	7500	2064354.81	1	448	12	5379.93
635	9	5715	1623823.71	1	458	9	4124.94
645	7	4515	1323141.02	1	468	7	3278.29
655	7	4585	1384708.05	1	478	7	3348.29
665	4	2660	827242.90	1	488	4	1953.31
675	11	7425	2376066.16	1	498	11	5481.60
685	7	4795	1577809.13	1	508	7	3558.29
695	6	4170	1409979.56	1	518	6	3109.96
705	3	2115	734375.65	1	528	3	1584.98
715	2	1430	509574.34	1	538	2	1076.65
725	4	2900	1059929.85	1	548	4	2193.31
735	1	735	275377.75	1	558	1	558.33
745	4	2980	1143892.16	1	568	4	2273.31
755	0	0	0.00	1	578	0	0.00
765	6	4590	1846581.72	1	588	6	3529.96
775	2	1550	637917.82	1	598	2	1196.65
785	0	0	0.00	1	608	0	0.00
795	1	795	341949.49	1	618	1	618.33
