# Supplementary material for: Exploring Explanations of Subglacial Bedform Sizes Using Statistical Models
Source: PLoS One. 2016 Jul 26;11(7):e0159489. doi: 10.1371/journal.pone.0159489 (PMC4961447; doi:10.1371/journal.pone.0159489)
Supplement: S1 File — Also includes a summary table of notation used in the manuscript. (ZIP) [file pone.0159489.s001.zip › S1 File/Hillier2013_worked_example.pdf]

Parameterisation: Worked Example

1. Plot of the data

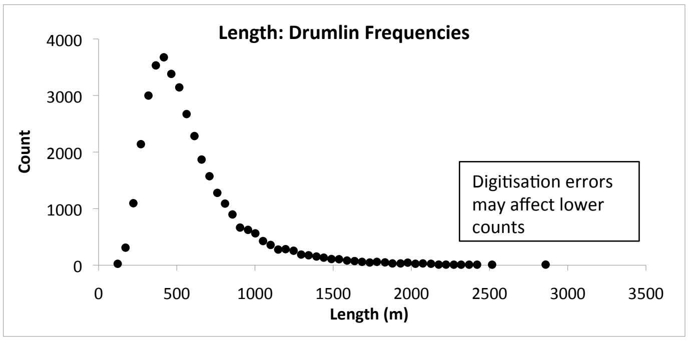

[1] These data are digitised from Fig.8 of Clark *et al* (2009). This is a marginally less straightforward than for using the raw data, but illustrates how evidence presented in the literature may be utilised. This also presents the opportunity for some considerations relating to digitised data to be raised.

If using raw data, they can be visualised in a histogram, but the binned data would play no part in the calcuation

2. Objective Parameterisation

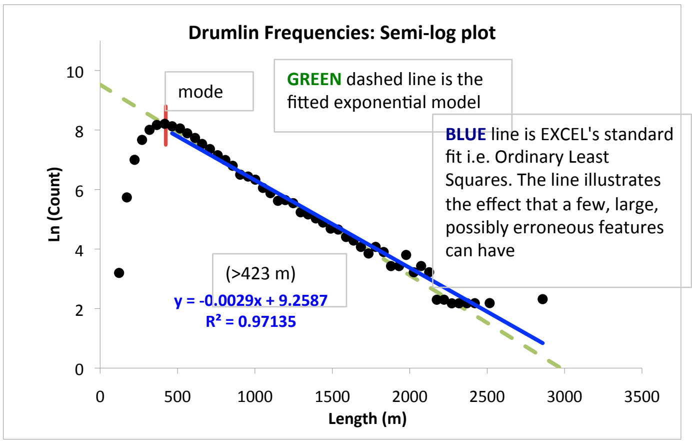

[2] Fit achieved. Details of the calculations are below. The difference between the fits illustrates the need for an objective, standardised procedure if comparisons are to b made.

Problem with OLS fitting: Should counts of zero be plotted? If so, upto what length and at what y value

3. Estimate of the mode

[3] Estimate the mode using the Gamma distribution and the method of moments.

Sum	36904
Mean	587.7
Standard Deviation	310.45
Alpha (α)	3.58
Lambda for Gamma (λ_g)	0.00610
**Mode (φ)**	**423.7**

Sequence of calculation

Formulae for digitised data. Simply use =AVERAGE() and = STDEV() when working with the raw observations

Mean

$$\bar{x} = \frac{1}{n}\sum c_j x_j$$

Standard Deviation

$$s_x = \sqrt{\frac{1}{n-1}\sum c_j (x_j - \bar{x})^2}$$

Alpha (α)

$$\hat{\alpha} = (\bar{x}/s_x)^2$$

Lambda for Gamma (λ_g)

$$\hat{\lambda}_g = \bar{x}/(s_x)^2$$

Mode (φ)

$$(\hat{\alpha} - 1)/\hat{\lambda}_g$$

NB: This is inside the range of 393-441 of the modal bin from the histogram of Clark *et al* (2009), but does not depend upon the selection of bins

Data: Here this is digitised from a histogram, but raw data should be used if available

For calcuations. Column D: Sum in mean. Column E: Sum in standard deviation

For plotting (above)

Length (*L*)	Count (*C*)	*C*L*	*C*(L-mean)*^{2}	Ln(count)
122	24	2995	5302378.46	3.20
172	310	53409	53407383.4	5.74
222	1096	243731	146132085	7.00
271	2140	579262	215018651	7.67
319	2999	956578	216576288	8.01
366	3532	1294201	172977506	8.17
417	3675	1534197	106561956	8.21
465	3382	1572634	50919011.1	8.13
516	3143	1622243	16122533	8.05
563	2672	1503766	1655900.09	7.89
613	2283	1399618	1465439.87	7.73

659	1867	1230436	9458623.94	7.53
709	1573	1115376	23175572.4	7.36
759	1278	970731	37644288.6	7.15
809	1089	880353	53131925.7	6.99
856	897	767683	64626520.8	6.80
905	665	601563	66787591.2	6.50
956	624	596588	84523799.3	6.44
1002	563	563885	96713446.8	6.33
1052	426	448397	91768169.6	6.06
1100	358	393502	93898073.4	5.88
1148	276	317250	86864208	5.62
1196	283	338746	104799345	5.65
1247	255	318383	110988867	5.54
1295	188	243293	94073764.8	5.24
1343	175	235041	99836641.3	5.16
1393	153	213024	99197345.3	5.03
1440	133	191683	96662196.2	4.89
1489	109	161863	88317033	4.69
1538	105	161776	94919712	4.66
1589	82	130185	82090663.3	4.41
1637	73	118930	79984949	4.29
1684	59	98875	70607895.3	4.07
1734	47	81609	61825128.3	3.85
1780	59	104503	83415630.6	4.07
1831	49	90524	76459869.9	3.90
1879	31	57900	51379847.6	3.43
1931	31	59529	55622785.8	3.43
1977	45	88580	86453695.3	3.80
2026	25	50723	51790223.5	3.22
2073	31	64011	68148853.5	3.43
2125	25	53275	59268391.8	3.22
2175	10	21630	25049649	2.30
2221	10	22124	26579890.1	2.30
2272	9	20012	24978335.8	2.18
2318	9	20455	26421924.6	2.18
2369	9	20940	28051002	2.18
2420	9	21427	29731259	2.18
2516	9	22347	33029328.3	2.18
2859	10	29000	52314147.6	2.32

Sum of the digitised data is 36904, differing only slightly from the 37,043 quote in Clark *et al* (2009).

4. Estimate of the gradient and intercept

[4] Estimate the gradient and intercept using a maximum likelihood estimator (MLE).

Sum (i.e. n_ϕ)	23128
Mean (k)	312.4
**Gradient (λ)**	**0.00320**

Mean calculated as above, and nf is the number of observations of size greater than the mode.

Gradient (λ)

$$\hat{\lambda} = 1/\bar{k}$$

Shift the data left, effectively creating an exponential starting from zero at the mode.

L above mode	Count (from above)	*C*L above mode*
41	3382	139645.334
92	3143	290369.174
139	2672	371655.834
189	2283	432251.358
235	1867	439160.33
285	1573	448887.886
336	1278	429035.035
385	1089	419052.099
432	897	387764.953
481	665	319798.345
532	624	332080.725
579	563	325514.88
628	426	267733.134
676	358	241934.537
725	276	200204.699
772	283	218735.876
823	255	210204.44
872	188	163717.573

919	175	160885.425
969	153	148234.673
1016	133	135273.694
1065	109	115805.414
1114	105	117192.668
1165	82	95461.1877
1213	73	88145.793
1261	59	74003.8709
1310	47	61664.907
1356	59	79620.4889
1408	49	69580.8517
1455	31	44843.4705
1507	31	46466.4313
1553	45	69592.3514
1602	25	40114.9774
1650	31	50930.287
1702	25	42654.0563
1751	10	17415.4393
1798	10	17903.7357
1848	9	16279.2763
1894	9	16715.9059
1945	9	17195.2452
1997	9	17676.0322
2092	9	18583.7593
2435	10	24701.3605

W	47.59	Estimate of bin width from digitisation in range 100-1950 m. With raw data, this would be known exactly because it is set when creating the plot
ln(n_ϕ)	10.05	
ln(λ)	-5.74	
ln(W)	3.86	
**x-intercept** (shifted back)	2975	$x_0 = [\ln(n_\phi \hat{\lambda} w_b)/\hat{\lambda}] + \phi$
**y-intercept** (shifted back)	9.5	$\hat{\lambda} x_0$
