# Supplementary material for: Exploring Explanations of Subglacial Bedform Sizes Using Statistical Models
Source: PLoS One. 2016 Jul 26;11(7):e0159489. doi: 10.1371/journal.pone.0159489 (PMC4961447; doi:10.1371/journal.pone.0159489)
Supplement: S1 File — Also includes a summary table of notation used in the manuscript. (ZIP) [file pone.0159489.s001.zip › S1 File/README.docx]

**README file describing the files accompanying Hillier et al. [2016]**

(1) Digitized frequencies: Files contained counts of bedform sizes as digitized from published papers, specifically to illustrate that the methods in Hillier et al. [2016] can be applied to published data even if the metrics for individual bedforms are not publically available. Deliberately, these were examined by the lead author prior to obtaining the actual frequencies used in the plots.

All sheets contain fully worked examples of how to calculate population parameters for the log-normal (μ, σ) and gamma (α, β) distributions as well as the exponent over the mode (φ, λ).

- **Clark_2009_Brit_L_counts_digitzed.xls**: Lengths of British drumlins digitized from Fig. 8 of Clark et al. (2009). From these data, bin widths were inferred to be 50 m, starting at 0.0.
- **Clark_2009_Brit_W_counts_digitized.xls**: Widths of British drumlins digitized from Fig. 8 of Clark et al. (2009) From these data, bin widths were inferred to be ~10 m.
- **Spagnolo_2012_H_counts_digitized.xls**: Heights of British drumlins digitized from Fig.5 of Spagnolo et al. (2012). From these data, bin widths were inferred to be 0.5 m, starting at 0.0.

(2) Actual frequencies: Files contain counts of bedform sizes as used to create the plots in the publications Clark et al. (2009) and Spagnolo et al. (2012).

All sheets contain fully worked examples of how to calculate population parameters.

- **Clark_2009_Brit_L_counts_original.xls**: Lengths of British drumlins digitized from Fig. 8 of Clark et al. (2009). Bin widths are 50 m, starting at 0 m.
- **Clark_2009_Brit_W_counts_original.xls**: Widths of British drumlins digitized from Fig. 8 of Clark et al. (2009) From these data, bin widths were inferred to be ~10 m.
- **Spagnolo_2012_H_counts_original.xls**: Heights of British drumlins digitized from Fig.5 of Spagnolo et al. (2012). Bin widths are 0.5 m, starting at 0.0 m.

(3) Raw individual data:

- **HS2012_HWL.txt**: This file contains the sizes (H, W, L) triplets of bedforms from the Loch Lomond study area used by Hillier and Smith (2012) and subsequent papers. These are the drumlins digitised from the NEXTmap DEM by Smith, and quantified by Method 2 of Hillier and Smith (2012). These are the data used in later analyses e.g. Hillier and Smith (2014) and Hillier et al. (2015). These sizes are used to create the plots in the publications.
- **HS2012_HWL_counts.xlsx**: Contains fully worked examples of how to calculate population parameters from freq**u**ency data.
- **HS2012_HWL_individual.xlsx**: Contains fully worked examples of how to calculate population parameters from the individual data for each drumlin.
- Despite Clark and Spagnolo being co-authors on this PLOS ONE publication, they are not the workers who did the mapping (i.e., generated the data) used in Clark et al. (2009) and Spagnolo et al. (2012) and so we are not in a position to make these data available.

(4) Worked examples of how the population parameters are calculated for each of the different types of data in the EXCEL sheets. The equations for calculating parameters of the log-normal distribution from counts or frequencies are given in Appendix B of Hillier et al. (2016), with other equations for these calculations given in Hillier et al. (2013) for the gamma distribution and Fowler et al. (2013) for the log-normal distribution. Equations for the exponent over a mode approximation were originally given in Hillier et al. (2013). The worked EXCEL example supplied with Hillier et al. (2013) is also provided here as **Hillier2013_worked_example.xls** and **Hillier2013_worked_example.pdf**.

(5) Table summarizing the notation used in the manuscript. **Table_of_Notation.docx**.
