# Supplementary material for: Exploring Explanations of Subglacial Bedform Sizes Using Statistical Models
Source: PLoS One. 2016 Jul 26;11(7):e0159489. doi: 10.1371/journal.pone.0159489 (PMC4961447; doi:10.1371/journal.pone.0159489)
Supplement: S1 File — Also includes a summary table of notation used in the manuscript. (ZIP) [file pone.0159489.s001.zip › S1 File/Spagnolo_2012_H_counts_digitized.xlsx.pdf]

Heights of British drumlins digitised from Fig.5 of Spagnolo et al. (2012)

Centre of bin	Count
0.26	0
0.75	161.309
1.25	608.535
1.73	1109.53
2.23	1361.31
2.74	1492.75
3.24	1507.25
3.76	1537.98
4.23	1455.19
4.73	1389.47
5.24	1408.25
5.74	1305.83
6.23	1225.6
6.75	1145.38
7.25	1048.08
7.74	977.24
8.23	867.994
8.73	744.239
9.23	653.77
9.74	611.095
10.25	551.351
10.75	506.117
11.24	470.27
11.74	369.559
12.24	344.808
12.74	312.376
13.23	279.943
13.71	250.071
14.23	184.353
14.74	209.104
15.23	180.085
15.74	152.774
16.22	135.704
16.73	128.876
17.25	125.462
17.75	98.1508
18.24	99.8577
18.73	96.4438
19.23	75.1067
19.73	79.3741
20.23	69.9858
20.72	56.33
21.22	46.9417
21.74	46.0882
22.25	39.2603
22.71	42.6743
23.23	33.2859
23.72	28.165
24.23	40.1138
24.74	29.0185
25.25	28.165
25.75	14.5092
26.23	20.4836
26.72	17.9232
27.23	16.2162
27.73	13.6558
28.24	17.9232
28.71	5.12091
29.25	13.6558
29.73	11.0953
30.24	8.53485
30.75	11.0953
31.23	5.12091
31.74	11.0953
32.23	3.41394
32.74	1.70697
33.21	7.68137
33.77	3.41394

**log-normal**		
μ	1.74	Parameters calculated in Sheet 'Calculation - log-normal'. Note that these are very close to the values of 1.74 and 0.69 obtained from the frequencies used to Fig 5 in Spagnolo et al [2012].
σ	0.68	
**Gamma**		
α	1.99	Parameters calculated in Sheet 'Calculation - rest'. α is close to the value of 2.03 obtained by frequency used to create Fig 5 in Spagnolo et al [2012] . β is close to the value obtained from the frequencies used to create Fig. 5 in Spagnolo et al [2012](i.e. to 0.29).
β (m^{-1})	0.29	
**Exponential tail**		
φ (m)	3.4	Mode is close to the value of 3.6 calculated for frequencies use to create the figure in Fig 5 in Spagnolo et al [2012], and λ is also close (i.e. to 0.20)
λ (m^{-1})	0.19	

NB: parameters differ very slightly from Hillier et al. [2013] who used some extra data cleaning steps to try and improve the digitized values

The effect of digitizing a published figure, as compared to the frequencies originally used to create it, is <7%. This is easily insufficient to alter the conclusions of Hillier et al. [2016], and small enough to suggest that using parameters obtained by digitizing previous figures will be useful in compilations and comparisons.

Heights of British drumlins digitised from Fig.5 of Spagnolo et al. (2012)

Parameter	Value		
**n**	25692.5		
**Mean of ln(xj)**	1.74	i.e. μ	=SUM(E28:E95)/C6
**Stdev of ln(xj)**	0.68	i.e. σ	=SQRT((1/(C6-1))*SUM(G28:G95))

Parameters μ and σ of the log-normal distribution are calculated according to the equations below; see Appendix B of Hillier et al. [2016]. Columns D to G are used for stages of the calculation, with formulae used in the top row of the table explicitly shown.

$$\hat{\mu} = \bar{x} = \frac{1}{n}\sum c_j \ln(x_j)$$

$$\hat{\sigma} = s_x = \sqrt{\frac{1}{n-1}\sum c_j\left[ln(x_j) - \overline{ln\ (x)}\right]^2}$$

Centre of bin j i.e. (xj)	Count (Cj)	ln(xj) =LN(B28)	cj*ln(xj) =C28*D28	ln(xj) - mean of ln(x) =D28-C7	cj*{[ln(xj) - mean of ln(x)]^2} =C28*(F28^2)
0.26	0	-1.35	0.00	-3.09	0.00
0.75	161.309	-0.28	-45.52	-2.02	660.89
1.25	608.535	0.22	135.63	-1.52	1404.16
1.73	1109.53	0.55	611.02	-1.19	1574.41
2.23	1361.31	0.80	1091.80	-0.94	1202.59
2.74	1492.75	1.01	1502.66	-0.74	807.04
3.24	1507.25	1.18	1773.14	-0.57	482.02
3.76	1537.98	1.32	2036.86	-0.42	268.14
4.23	1455.19	1.44	2100.00	-0.30	129.93
4.73	1389.47	1.55	2158.96	-0.19	49.17
5.24	1408.25	1.66	2331.37	-0.09	10.51
5.74	1305.83	1.75	2282.36	0.01	0.05
6.23	1225.6	1.83	2241.47	0.09	9.27
6.75	1145.38	1.91	2187.96	0.17	32.45
7.25	1048.08	1.98	2076.29	0.24	59.93
7.74	977.24	2.05	1999.21	0.30	90.23
8.23	867.994	2.11	1829.62	0.37	116.24
8.73	744.239	2.17	1612.27	0.42	134.06
9.23	653.77	2.22	1453.16	0.48	151.14
9.74	611.095	2.28	1390.93	0.53	174.40
10.25	551.351	2.33	1282.89	0.58	188.62
10.75	506.117	2.38	1202.06	0.63	202.88
11.24	470.27	2.42	1137.66	0.68	215.69
11.74	369.559	2.46	910.31	0.72	192.28
12.24	344.808	2.50	863.60	0.76	200.55
12.74	312.376	2.55	795.03	0.80	201.52
13.23	279.943	2.58	722.94	0.84	197.78
13.71	250.071	2.62	654.79	0.88	192.12
14.23	184.353	2.66	489.54	0.91	153.85
14.74	209.104	2.69	562.57	0.95	188.12
15.23	180.085	2.72	490.46	0.98	173.51
15.74	152.774	2.76	421.07	1.01	157.16
16.22	135.704	2.79	378.14	1.04	148.08
16.73	128.876	2.82	363.08	1.08	149.02
17.25	125.462	2.85	357.28	1.11	153.41
17.75	98.1508	2.88	282.34	1.13	126.37
18.24	99.8577	2.90	289.94	1.16	134.75
18.73	96.4438	2.93	282.62	1.19	136.22
19.23	75.1067	2.96	222.05	1.21	110.79
19.73	79.3741	2.98	236.69	1.24	122.05
20.23	69.9858	3.01	210.47	1.27	112.05
20.72	56.33	3.03	170.73	1.29	93.60
21.22	46.9417	3.06	143.41	1.31	80.95
21.74	46.0882	3.08	141.91	1.34	82.42
22.25	39.2603	3.10	121.79	1.36	72.64
22.71	42.6743	3.12	133.26	1.38	81.37
23.23	33.2859	3.15	104.69	1.40	65.56
23.72	28.165	3.17	89.18	1.42	57.15
24.23	40.1138	3.19	127.86	1.45	83.83
24.74	29.0185	3.21	93.10	1.47	62.39
25.25	28.165	3.23	90.94	1.49	62.28
25.75	14.5092	3.25	47.13	1.51	32.93
26.23	20.4836	3.27	66.92	1.53	47.64
26.72	17.9232	3.29	58.88	1.54	42.69
27.23	16.2162	3.30	53.59	1.56	39.59
27.73	13.6558	3.32	45.37	1.58	34.12
28.24	17.9232	3.34	59.87	1.60	45.81
28.71	5.12091	3.36	17.19	1.62	13.36
29.25	13.6558	3.38	46.10	1.63	36.46
29.73	11.0953	3.39	37.64	1.65	30.22
30.24	8.53485	3.41	29.10	1.67	23.72
30.75	11.0953	3.43	38.01	1.68	31.46
31.23	5.12091	3.44	17.62	1.70	14.79
31.74	11.0953	3.46	38.36	1.72	32.66
32.23	3.41394	3.47	11.86	1.73	10.23
32.74	1.70697	3.49	5.95	1.75	5.21
33.21	7.68137	3.50	26.91	1.76	23.82
33.77	3.41394	3.52	12.02	1.78	10.79

Heights of British drumlins digitised from Fig.5 of Spagnolo et al. (2012)

Parameters α and β of the gamma distribution, and mode ϕ and gradient above it λ are calculated according to the equations below; see Hillier et al. [2013]. Columns D to I are used for stages of the calculation, with formulae used in the top row of the table explicitly shown. Similarly, flormulae used for the parameters are shown explicitly.

Parameter	Value	
n	25692	=SUM(C33:C114)
Mean	6.84	=SUM(D33:D114)/C14
Standard Deviation	4.84	=SQRT((1/(C14-1))*SUM(E35:E102))
Alpha (α)	1.99	=(C15/C16)^2
Beta (β)	0.2915	=C15/(C16^2)
Mode (ϕ)	3.41	=(C17-1)/C18
Exponent (λ)	0.1948	=SUM(H35:H114)/SUM(I35:I102)

Sequence of calculation ↓

Alpha (α) $\hat{\alpha} = (\bar{x}/s_x)^2$

Mean $\bar{x} = \frac{1}{n}\sum c_j x_j$

β - Called lambda for Gamma (λ_g) in Hillier et al. [2013]

$\hat{\lambda}_g = \bar{x}/(s_x)^2$

Gradient (λ)

$\hat{\lambda} = 1/\bar{k}$

k bar is the mean of values exceeding the mode. That is, it is only calculated for a value over the mode, and then only includes the amount by which it is over the mode.

Standard Deviation

$s_x = \sqrt{\frac{1}{n-1}\sum c_j (x_j - \bar{x})^2}$

Mode (φ)

$(\hat{\alpha} - 1)/\hat{\lambda}_g$

Centre of bin j i.e. (xj)	Count (Cj)	xj*Cj<br>=B35*C35	Cj*(xj - mean x)^2<br>=C35*((B35-C$15)^2	Above mode?<br>=IF(B35-C$19 > 0, 1, 0)	Amount above mode<br>=(B35-C$19)*F35	Cj sbove mode<br>=F35*C35	xj*Cj above mode<br>=G35*H35
0.26	0	0	0.00	0	0.00	0	0.00
0.75	161.309	121.646182	5972.66	0	0.00	0	0.00
1.25	608.535	760.474019	19011.18	0	0.00	0	0.00
1.73	1109.53	1924.4465	28910.58	0	0.00	0	0.00
2.23	1361.31	3035.77575	28918.10	0	0.00	0	0.00
2.74	1492.75	4084.73125	25125.65	0	0.00	0	0.00
3.24	1507.25	4887.57465	19494.11	0	0.00	0	0.00
3.76	1537.98	5782.52796	14582.47	1	0.35	1538	540.72
4.23	1455.19	6161.04163	9876.45	1	0.83	1455	1201.40
4.73	1389.47	6571.35942	6183.93	1	1.32	1389	1835.71
5.24	1408.25	7373.23086	3619.99	1	1.83	1408	2573.57
5.74	1305.83	7498.18033	1571.32	1	2.33	1306	3047.59
6.23	1225.6	7631.65187	459.29	1	2.82	1226	3454.51
6.75	1145.38	7736.75556	8.14	1	3.35	1145	3833.02
7.25	1048.08	7598.91539	177.29	1	3.84	1048	4026.80
7.74	977.24	7559.0589	784.67	1	4.33	977	4228.39
8.23	867.994	7144.17218	1681.00	1	4.82	868	4185.84
8.73	744.239	6494.40813	2650.63	1	5.32	744	3957.86
9.23	653.77	6035.97729	3745.45	1	5.82	654	3807.77
9.74	611.095	5951.39921	5138.86	1	6.33	611	3868.64
10.25	551.351	5648.70127	6396.75	1	6.84	551	3769.56
10.75	506.117	5441.56754	7747.72	1	7.34	506	3716.60
11.24	470.27	5284.14183	9093.52	1	7.83	470	3681.35
11.74	369.559	4339.62047	8886.38	1	8.33	370	3080.07
12.24	344.808	4219.86375	10051.86	1	8.83	345	3044.67
12.74	312.376	3981.10717	10894.33	1	9.34	312	2916.45
13.23	279.943	3703.47792	11431.96	1	9.82	280	2749.36
13.71	250.071	3429.52371	11820.32	1	10.31	250	2577.22
14.23	184.353	2623.58285	10074.07	1	10.82	184	1995.26
14.74	209.104	3081.69111	13045.44	1	11.33	209	2369.01
15.23	180.085	2743.27082	12689.15	1	11.82	180	2129.50
15.74	152.774	2404.58637	12102.49	1	12.33	153	1883.90
16.22	135.704	2201.70241	11953.24	1	12.82	136	1739.19
16.73	128.876	2156.18569	12609.87	1	13.32	129	1716.94
17.25	125.462	2163.94348	13592.85	1	13.84	125	1736.34
17.75	98.1508	1742.57912	11693.55	1	14.35	98	1408.06
18.24	99.8577	1821.2946	12977.20	1	14.83	100	1480.96
18.73	96.4438	1806.82637	13647.00	1	15.33	96	1478.12
19.23	75.1067	1444.30184	11531.58	1	15.82	75	1188.32
19.73	79.3741	1565.70175	13181.14	1	16.32	79	1295.18
20.23	69.9858	1415.94571	12553.27	1	16.82	70	1177.42
20.72	56.33	1166.97171	10848.57	1	17.31	56	974.99
21.22	46.9417	996.248393	9712.30	1	17.81	47	836.26
21.74	46.0882	1001.96669	10233.64	1	18.33	46	844.89
22.25	39.2603	873.404264	9320.00	1	18.84	39	739.60
22.71	42.6743	969.124818	10748.85	1	19.30	43	823.68
23.23	33.2859	773.128271	8939.33	1	19.82	33	659.68
23.72	28.165	668.141396	8028.38	1	20.31	28	572.15
24.23	40.1138	971.909237	12130.57	1	20.82	40	835.19
24.74	29.0185	717.775499	9293.73	1	21.33	29	618.87
25.25	28.165	711.228213	9549.19	1	21.84	28	615.24
25.75	14.5092	373.57998	5187.64	1	22.34	15	324.13
26.23	20.4836	537.338085	7704.09	1	22.82	20	467.52
26.72	17.9232	478.861304	7082.34	1	23.31	18	417.77
27.23	16.2162	441.640099	6745.53	1	23.83	16	386.37
27.73	13.6558	378.675334	5959.83	1	24.32	14	332.13
28.24	17.9232	506.086644	8206.09	1	24.83	18	445.00
28.71	5.12091	147.023374	2449.62	1	25.30	5	129.57
29.25	13.6558	399.41986	6858.09	1	25.84	14	352.88
29.73	11.0953	329.905431	5815.83	1	26.33	11	292.09
30.24	8.53485	258.095571	4673.81	1	26.83	9	229.01
30.75	11.0953	341.141641	6341.71	1	27.34	11	303.33
31.23	5.12091	159.932676	3046.85	1	27.82	5	142.48
31.74	11.0953	352.138193	6878.40	1	28.33	11	314.32
32.23	3.41394	110.042211	2201.53	1	28.82	3	98.41
32.74	1.70697	55.8853443	1145.09	1	29.33	2	50.07
33.21	7.68137	255.125951	5343.30	1	29.81	8	228.95
33.77	3.41394	115.301727	2476.75	1	30.37	3	103.67
