# Supplementary material for: Exploring Explanations of Subglacial Bedform Sizes Using Statistical Models
Source: PLoS One. 2016 Jul 26;11(7):e0159489. doi: 10.1371/journal.pone.0159489 (PMC4961447; doi:10.1371/journal.pone.0159489)
Supplement: S1 File — Also includes a summary table of notation used in the manuscript. (ZIP) [file pone.0159489.s001.zip › S1 File/Spagnolo_2012_H_counts_original.xlsx.pdf]

**Heights of British drumlins: the original frequencies input into Fig.5 of Spagnolo et al. (2012)**

| Centre of bin | Count |
|---|---|
| 0.25 | 1 |
| 0.75 | 162 |
| 1.25 | 610 |
| 1.75 | 1111 |
| 2.25 | 1363 |
| 2.75 | 1494 |
| 3.25 | 1508 |
| 3.75 | 1539 |
| 4.25 | 1457 |
| 4.75 | 1392 |
| 5.25 | 1410 |
| 5.75 | 1308 |
| 6.25 | 1227 |
| 6.75 | 1147 |
| 7.25 | 1051 |
| 7.75 | 981 |
| 8.25 | 871 |
| 8.75 | 747 |
| 9.25 | 655 |
| 9.75 | 612 |
| 10.25 | 553 |
| 10.75 | 508 |
| 11.25 | 473 |
| 11.75 | 370 |
| 12.25 | 347 |
| 12.75 | 315 |
| 13.25 | 280 |
| 13.75 | 252 |
| 14.25 | 186 |
| 14.75 | 209 |
| 15.25 | 182 |
| 15.75 | 154 |
| 16.25 | 138 |
| 16.75 | 130 |
| 17.25 | 127 |
| 17.75 | 101 |
| 18.25 | 102 |
| 18.75 | 97 |
| 19.25 | 77 |
| 19.75 | 81 |
| 20.25 | 72 |
| 20.75 | 58 |
| 21.25 | 48 |
| 21.75 | 46 |
| 22.25 | 39 |
| 22.75 | 43 |
| 23.25 | 34 |
| 23.75 | 28 |
| 24.25 | 41 |
| 24.75 | 31 |
| 25.25 | 30 |
| 25.75 | 16 |
| 26.25 | 22 |
| 26.75 | 20 |
| 27.25 | 17 |
| 27.75 | 15 |
| 28.25 | 19 |
| 28.75 | 7 |
| 29.25 | 16 |
| 29.75 | 13 |
| 30.25 | 10 |
| 30.75 | 13 |
| 31.25 | 6 |
| 31.75 | 11 |
| 32.25 | 5 |
| 32.75 | 2 |
| 33.25 | 7 |
| 33.75 | 3 |
| 34.25 | 3 |
| 34.75 | 3 |
| 35.25 | 3 |
| 35.75 | 3 |
| 36.25 | 1 |
| 36.75 | 5 |
| 37.25 | 5 |
| 37.75 | 1 |
| 38.25 | 5 |
| 38.75 | 4 |
| 39.25 | 0 |
| 39.75 | 0 |

**log-normal**

| | |
|---|---|
| μ | 1.74 |
| σ | 0.69 |

Parameters calculated in Sheet 'Calculation - log-normal'. Note that these are very close to the values of 1.78 and 0.68 obtained by digitizing Fig 5 in Spagnolo et al [2012]

**Gamma**

| | |
|---|---|
| α | 2.03 |
| β ($m^{-1}$) | 0.29 |

Parameters calculated in Sheet 'Calculation - rest'. α is close to the value obtained by digitizing Fig 5 in Spagnolo et al [2012] (i.e., 1.99). β is also close (i.e. to 0.29)

**Exponential tail**

| | |
|---|---|
| φ (m) | 3.6 |
| λ ($m^{-1}$) | 0.20 |

Mode of is close to the 3.4 calculated for digitized values, and λ is also close (i.e. to 0.19)

**The effect of digitizing a published figure, as compared to the frequencies originally used to create it, is always <7%. This is easily insufficient to alter the conclusions of Hillier et al. [2016], and small enough to suggest that using parameters obtained by digitizing previous figures will be useful in compilations and comparisons.**

More 33 — 33 drumlins have H > 40, not included in plot in Spagnolo (2012)

**Heights of British drumlins: the original frequencies input into Fig.5 of Spagnolo et al. (2012)**

| Parameter | Value | | |
|---|---|---|---|
| n | 26033 | | |
| Mean of ln(xj) | 1.74 | i.e. μ | =SUM(E28:E107)/C6 |
| Stdev of ln(xj) | 0.69 | i.e. σ | =SQRT((1/(C6-1))*SUM(G28:G107)) |

Parameters μ and σ of the log-normal distribution are calculated according to the equations below; see Appendix B of Hillier et al. [2016]. Columns D to G are used for stages of the calculation, with formulae used in the top row of the table explicitly shown.

$$\hat{\mu} = \bar{x} = \frac{1}{n}\sum c_j \ln(x_j)$$

$$\hat{\sigma} = s_x = \sqrt{\frac{1}{n-1}\sum c_j\left[ln(x_j) - \overline{ln\,(x)}\right]^2}$$

| Centre of bin j i.e. (xj) | Count (Cj) | ln(xj) =LN(B28) | cj*ln(xj) =C28*D28 | ln(xj) - mean of ln(x) =D28-$C$7 | cj*{[ln(xj) - mean of ln(x)]^2} =C28*(F28^2) |
|---|---|---|---|---|---|
| 0.25 | 1 | -1.39 | -1.39 | -3.12 | 9.74 |
| 0.75 | 162 | -0.29 | -46.60 | -2.02 | 662.87 |
| 1.25 | 610 | 0.22 | 136.12 | -1.51 | 1394.53 |
| 1.75 | 1111 | 0.56 | 621.73 | -1.18 | 1535.22 |
| 2.25 | 1363 | 0.81 | 1105.30 | -0.92 | 1164.21 |
| 2.75 | 1494 | 1.01 | 1511.33 | -0.72 | 782.11 |
| 3.25 | 1508 | 1.18 | 1777.41 | -0.56 | 466.98 |
| 3.75 | 1539 | 1.32 | 2034.18 | -0.41 | 262.98 |
| 4.25 | 1457 | 1.45 | 2108.16 | -0.29 | 121.03 |
| 4.75 | 1392 | 1.56 | 2168.94 | -0.18 | 43.60 |
| 5.25 | 1410 | 1.66 | 2338.10 | -0.08 | 8.34 |
| 5.75 | 1308 | 1.75 | 2287.95 | 0.01 | 0.26 |
| 6.25 | 1227 | 1.83 | 2248.58 | 0.10 | 11.65 |
| 6.75 | 1147 | 1.91 | 2190.25 | 0.17 | 34.89 |
| 7.25 | 1051 | 1.98 | 2082.03 | 0.25 | 63.53 |
| 7.75 | 981 | 2.05 | 2008.79 | 0.31 | 95.84 |
| 8.25 | 871 | 2.11 | 1838.00 | 0.38 | 122.54 |
| 8.75 | 747 | 2.17 | 1620.28 | 0.43 | 140.65 |
| 9.25 | 655 | 2.22 | 1457.13 | 0.49 | 156.94 |
| 9.75 | 612 | 2.28 | 1393.69 | 0.54 | 179.87 |
| 10.25 | 553 | 2.33 | 1286.98 | 0.59 | 193.90 |
| 10.75 | 508 | 2.37 | 1206.45 | 0.64 | 207.93 |
| 11.25 | 473 | 2.42 | 1144.83 | 0.69 | 222.10 |
| 11.75 | 370 | 2.46 | 911.63 | 0.73 | 196.48 |
| 12.25 | 347 | 2.51 | 869.42 | 0.77 | 205.95 |
| 12.75 | 315 | 2.55 | 801.84 | 0.81 | 206.87 |
| 13.25 | 280 | 2.58 | 723.52 | 0.85 | 201.76 |
| 13.75 | 252 | 2.62 | 660.50 | 0.89 | 197.78 |
| 14.25 | 186 | 2.66 | 494.16 | 0.92 | 157.99 |
| 14.75 | 209 | 2.69 | 562.47 | 0.96 | 191.06 |
| 15.25 | 182 | 2.72 | 495.87 | 0.99 | 178.18 |
| 15.75 | 154 | 2.76 | 424.55 | 1.02 | 160.76 |
| 16.25 | 138 | 2.79 | 384.76 | 1.05 | 153.00 |
| 16.75 | 130 | 2.82 | 366.39 | 1.08 | 152.55 |
| 17.25 | 127 | 2.85 | 361.67 | 1.11 | 157.23 |
| 17.75 | 101 | 2.88 | 290.51 | 1.14 | 131.55 |
| 18.25 | 102 | 2.90 | 296.22 | 1.17 | 139.40 |
| 18.75 | 97 | 2.93 | 284.33 | 1.20 | 138.76 |
| 19.25 | 77 | 2.96 | 227.73 | 1.22 | 115.05 |
| 19.75 | 81 | 2.98 | 241.64 | 1.25 | 126.16 |
| 20.25 | 72 | 3.01 | 216.59 | 1.27 | 116.68 |
| 20.75 | 58 | 3.03 | 175.89 | 1.30 | 97.63 |
| 21.25 | 48 | 3.06 | 146.71 | 1.32 | 83.79 |
| 21.75 | 46 | 3.08 | 141.66 | 1.34 | 83.15 |
| 22.25 | 39 | 3.10 | 120.99 | 1.37 | 72.90 |
| 22.75 | 43 | 3.12 | 134.36 | 1.39 | 83.01 |
| 23.25 | 34 | 3.15 | 106.97 | 1.41 | 67.71 |
| 23.75 | 28 | 3.17 | 88.69 | 1.43 | 57.45 |
| 24.25 | 41 | 3.19 | 130.73 | 1.45 | 86.59 |
| 24.75 | 31 | 3.21 | 99.47 | 1.47 | 67.32 |
| 25.25 | 30 | 3.23 | 96.86 | 1.49 | 66.93 |
| 25.75 | 16 | 3.25 | 51.97 | 1.51 | 36.64 |
| 26.25 | 22 | 3.27 | 71.89 | 1.53 | 51.67 |
| 26.75 | 20 | 3.29 | 65.73 | 1.55 | 48.14 |
| 27.25 | 17 | 3.31 | 56.19 | 1.57 | 41.90 |
| 27.75 | 15 | 3.32 | 49.85 | 1.59 | 37.83 |
| 28.25 | 19 | 3.34 | 63.48 | 1.61 | 49.00 |
| 28.75 | 7 | 3.36 | 23.51 | 1.62 | 18.45 |
| 29.25 | 16 | 3.38 | 54.01 | 1.64 | 43.07 |
| 29.75 | 13 | 3.39 | 44.11 | 1.66 | 35.72 |
| 30.25 | 10 | 3.41 | 34.09 | 1.67 | 28.03 |
| 30.75 | 13 | 3.43 | 44.54 | 1.69 | 37.16 |
| 31.25 | 6 | 3.44 | 20.65 | 1.71 | 17.48 |
| 31.75 | 11 | 3.46 | 38.04 | 1.72 | 32.65 |
| 32.25 | 5 | 3.47 | 17.37 | 1.74 | 15.11 |
| 32.75 | 2 | 3.49 | 6.98 | 1.75 | 6.15 |
| 33.25 | 7 | 3.50 | 24.53 | 1.77 | 21.90 |
| 33.75 | 3 | 3.52 | 10.56 | 1.78 | 9.55 |
| 34.25 | 3 | 3.53 | 10.60 | 1.80 | 9.70 |
| 34.75 | 3 | 3.55 | 10.64 | 1.81 | 9.86 |
| 35.25 | 3 | 3.56 | 10.69 | 1.83 | 10.02 |
| 35.75 | 3 | 3.58 | 10.73 | 1.84 | 10.17 |
| 36.25 | 1 | 3.59 | 3.59 | 1.86 | 3.44 |
| 36.75 | 5 | 3.60 | 18.02 | 1.87 | 17.47 |
| 37.25 | 5 | 3.62 | 18.09 | 1.88 | 17.72 |
| 37.75 | 1 | 3.63 | 3.63 | 1.90 | 3.59 |
| 38.25 | 5 | 3.64 | 18.22 | 1.91 | 18.22 |
| 38.75 | 4 | 3.66 | 14.63 | 1.92 | 14.78 |
| 39.25 | 0 | 3.67 | 0.00 | 1.93 | 0.00 |
| 39.75 | 0 | 3.68 | 0.00 | 1.95 | 0.00 |

**Heights of British drumlins: the original frequencies input into Fig.5 of Spagnolo et al. (2012)**

Parameters α and β of the gamma distribution, and mode ϕ and gradient above it λ are calculated according to the equations below; see Hillier et al. [2013]. Columns D to I are used for stages of the calculation, with formulae used in the top row of the table explicitly shown. Similarly, flormulae used for the parameters are shown explicitly.

| Parameter | Value | Formula |
|---|---|---|
| n | 26033 | =SUM(C33:C114) |
| Mean | 7.09 | =SUM(D33:D114)/C14 |
| Standard Deviation | 4.98 | =SQRT((1/(C14-1))*SUM(E35:E114)) |
| Alpha (α) | 2.03 | =(C15/C16)^2 |
| Beta (β) | 0.2860 | =C15/(C16^2) |
| Mode (ϕ) | 3.60 | =(C17-1)/C18 |
| Exponent (λ) | 0.2007 | =SUM(H35:H114)/SUM(I35:I114) |

Sequence of calculation ↓

Alpha (α) $$\hat{\alpha} = (\bar{x}/s_x)^2$$

Mean $$\bar{x} = \frac{1}{n}\sum c_j x_j$$

β - Called lambda for Gamma ($\lambda_g$) in Hillier et al. [2013]

$$\hat{\lambda}_g = \bar{x}/(s_x)^2$$

Standard Deviation

$$s_x = \sqrt{\frac{1}{n-1}\sum c_j (x_j - \bar{x})^2}$$

Mode (φ)

$$(\hat{\alpha} - 1)/\hat{\lambda}_g$$

Gradient (λ)

$$\hat{\lambda} = 1/\bar{k}$$

k bar is the mean of values exceeding the mode. That is, it is only calculated for a value over the mode, and then only includes the amount by which it is over the mode.

| Centre of bin j i.e. (xj) | Count (Cj) | xj*Cj<br>=B35*C35 | Cj*(xj - mean x)^2<br>=C35*((B35-C$15)^2 | Above mode?<br>=IF(B35-C$19 > 0, 1, 0) | Amount above mode<br>=(B35-C$19)*F35 | Cj sbove mode<br>=F35*C35 | xj*Cj above mode<br>=G35*H35 |
|---|---|---|---|---|---|---|---|
| 0.25 | 1 | 0.25 | 46.82 | 0 | 0.00 | 0 | 0.00 |
| 0.75 | 162 | 121.5 | 6516.44 | 0 | 0.00 | 0 | 0.00 |
| 1.25 | 610 | 762.5 | 20820.92 | 0 | 0.00 | 0 | 0.00 |
| 1.75 | 1111 | 1944.25 | 31708.31 | 0 | 0.00 | 0 | 0.00 |
| 2.25 | 1363 | 3066.75 | 31959.65 | 0 | 0.00 | 0 | 0.00 |
| 2.75 | 1494 | 4108.5 | 28170.42 | 0 | 0.00 | 0 | 0.00 |
| 3.25 | 1508 | 4901 | 22263.19 | 0 | 0.00 | 0 | 0.00 |
| 3.75 | 1539 | 5771.25 | 17192.28 | 1 | 0.15 | 1539 | 237.59 |
| 4.25 | 1457 | 6192.25 | 11770.75 | 1 | 0.65 | 1457 | 953.43 |
| 4.75 | 1392 | 6612 | 7637.13 | 1 | 1.15 | 1392 | 1606.90 |
| 5.25 | 1410 | 7402.5 | 4785.72 | 1 | 1.65 | 1410 | 2332.67 |
| 5.75 | 1308 | 7521 | 2356.77 | 1 | 2.15 | 1308 | 2817.93 |
| 6.25 | 1227 | 7668.75 | 870.55 | 1 | 2.65 | 1227 | 3256.92 |
| 6.75 | 1147 | 7742.25 | 134.41 | 1 | 3.15 | 1147 | 3618.07 |
| 7.25 | 1051 | 7619.75 | 26.13 | 1 | 3.65 | 1051 | 3840.75 |
| 7.75 | 981 | 7602.75 | 424.33 | 1 | 4.15 | 981 | 4075.45 |
| 8.25 | 871 | 7185.75 | 1167.34 | 1 | 4.65 | 871 | 4053.96 |
| 8.75 | 747 | 6536.25 | 2052.69 | 1 | 5.15 | 747 | 3850.32 |
| 9.25 | 655 | 6058.75 | 3049.42 | 1 | 5.65 | 655 | 3703.62 |
| 9.75 | 612 | 5967 | 4322.73 | 1 | 6.15 | 612 | 3766.48 |
| 10.25 | 553 | 5668.25 | 5513.95 | 1 | 6.65 | 553 | 3679.87 |
| 10.75 | 508 | 5461 | 6796.36 | 1 | 7.15 | 508 | 3634.42 |
| 11.25 | 473 | 5321.25 | 8176.44 | 1 | 7.65 | 473 | 3620.52 |
| 11.75 | 370 | 4347.5 | 8026.79 | 1 | 8.15 | 370 | 3017.12 |
| 12.25 | 347 | 4250.75 | 9230.79 | 1 | 8.65 | 347 | 3003.07 |
| 12.75 | 315 | 4016.25 | 10082.96 | 1 | 9.15 | 315 | 2883.63 |
| 13.25 | 280 | 3710 | 10616.78 | 1 | 9.65 | 280 | 2703.23 |
| 13.75 | 252 | 3465 | 11169.84 | 1 | 10.15 | 252 | 2558.90 |
| 14.25 | 186 | 2650.5 | 9529.24 | 1 | 10.65 | 186 | 1981.71 |
| 14.75 | 209 | 3082.75 | 12255.79 | 1 | 11.15 | 209 | 2331.27 |
| 15.25 | 182 | 2775.5 | 12111.70 | 1 | 11.65 | 182 | 2121.10 |
| 15.75 | 154 | 2425.5 | 11543.15 | 1 | 12.15 | 154 | 1871.77 |
| 16.25 | 138 | 2242.5 | 11573.12 | 1 | 12.65 | 138 | 1746.30 |
| 16.75 | 130 | 2177.5 | 12125.21 | 1 | 13.15 | 130 | 1710.07 |
| 17.25 | 127 | 2190.75 | 13103.68 | 1 | 13.65 | 127 | 1734.11 |
| 17.75 | 101 | 1792.75 | 11472.21 | 1 | 14.15 | 101 | 1429.59 |
| 18.25 | 102 | 1861.5 | 12698.38 | 1 | 14.65 | 102 | 1494.75 |
| 18.75 | 97 | 1818.75 | 13182.46 | 1 | 15.15 | 97 | 1469.97 |
| 19.25 | 77 | 1482.25 | 11381.32 | 1 | 15.65 | 77 | 1205.39 |
| 19.75 | 81 | 1599.75 | 12977.58 | 1 | 16.15 | 81 | 1308.50 |
| 20.25 | 72 | 1458 | 12464.98 | 1 | 16.65 | 72 | 1199.12 |
| 20.75 | 58 | 1203.5 | 10818.88 | 1 | 17.15 | 58 | 994.95 |
| 21.25 | 48 | 1020 | 9621.12 | 1 | 17.65 | 48 | 847.41 |
| 21.75 | 46 | 1000.5 | 9882.99 | 1 | 18.15 | 46 | 835.10 |
| 22.25 | 39 | 867.75 | 8960.46 | 1 | 18.65 | 39 | 727.52 |
| 22.75 | 43 | 978.25 | 10542.01 | 1 | 19.15 | 43 | 823.64 |
| 23.25 | 34 | 790.5 | 8876.41 | 1 | 19.65 | 34 | 668.25 |
| 23.75 | 28 | 665 | 7769.40 | 1 | 20.15 | 28 | 564.32 |
| 24.25 | 41 | 994.25 | 12069.83 | 1 | 20.65 | 41 | 846.83 |
| 24.75 | 31 | 767.25 | 9665.61 | 1 | 21.15 | 31 | 655.79 |
| 25.25 | 30 | 757.5 | 9891.05 | 1 | 21.65 | 30 | 649.63 |
| 25.75 | 16 | 412 | 5569.75 | 1 | 22.15 | 16 | 354.47 |
| 26.25 | 22 | 577.5 | 8074.37 | 1 | 22.65 | 22 | 498.40 |
| 26.75 | 20 | 535 | 7728.49 | 1 | 23.15 | 20 | 463.09 |
| 27.25 | 17 | 463.25 | 6907.65 | 1 | 23.65 | 17 | 402.12 |
| 27.75 | 15 | 416.25 | 6401.10 | 1 | 24.15 | 15 | 362.32 |
| 28.25 | 19 | 536.75 | 8505.30 | 1 | 24.65 | 19 | 468.43 |
| 28.75 | 7 | 201.25 | 3283.39 | 1 | 25.15 | 7 | 176.08 |
| 29.25 | 16 | 468 | 7855.41 | 1 | 25.65 | 16 | 410.47 |
| 29.75 | 13 | 386.75 | 6673.82 | 1 | 26.15 | 13 | 340.01 |
| 30.25 | 10 | 302.5 | 5362.78 | 1 | 26.65 | 10 | 266.54 |
| 30.75 | 13 | 399.75 | 7275.92 | 1 | 27.15 | 13 | 353.01 |
| 31.25 | 6 | 187.5 | 3501.56 | 1 | 27.65 | 6 | 165.93 |
| 31.75 | 11 | 349.25 | 6688.02 | 1 | 28.15 | 11 | 309.70 |
| 32.25 | 5 | 161.25 | 3164.55 | 1 | 28.65 | 5 | 143.27 |
| 32.75 | 2 | 65.5 | 1316.63 | 1 | 29.15 | 2 | 58.31 |
| 33.25 | 7 | 232.75 | 4789.57 | 1 | 29.65 | 7 | 207.58 |
| 33.75 | 3 | 101.25 | 2131.90 | 1 | 30.15 | 3 | 90.46 |
| 34.25 | 3 | 102.75 | 2212.62 | 1 | 30.65 | 3 | 91.96 |
| 34.75 | 3 | 104.25 | 2294.84 | 1 | 31.15 | 3 | 93.46 |
| 35.25 | 3 | 105.75 | 2378.57 | 1 | 31.65 | 3 | 94.96 |
| 35.75 | 3 | 107.25 | 2463.79 | 1 | 32.15 | 3 | 96.46 |
| 36.25 | 1 | 36.25 | 850.17 | 1 | 32.65 | 1 | 32.65 |
| 36.75 | 5 | 183.75 | 4397.89 | 1 | 33.15 | 5 | 165.77 |
| 37.25 | 5 | 186.25 | 4547.43 | 1 | 33.65 | 5 | 168.27 |
| 37.75 | 1 | 37.75 | 939.89 | 1 | 34.15 | 1 | 34.15 |
| 38.25 | 5 | 191.25 | 4854.01 | 1 | 34.65 | 5 | 173.27 |
| 38.75 | 4 | 155 | 4008.84 | 1 | 35.15 | 4 | 140.62 |
| 39.25 | 0 | 0 | 0.00 | 1 | 35.65 | 0 | 0.00 |
| 39.75 | 0 | 0 | 0.00 | 1 | 36.15 | 0 | 0.00 |
