# Supplementary material for: Exploring Explanations of Subglacial Bedform Sizes Using Statistical Models
Source: PLoS One. 2016 Jul 26;11(7):e0159489. doi: 10.1371/journal.pone.0159489 (PMC4961447; doi:10.1371/journal.pone.0159489)
Supplement: S1 File — Also includes a summary table of notation used in the manuscript. (ZIP) [file pone.0159489.s001.zip › S1 File/Table_of_Notation.docx]

**Table of Notation**

| Symbol | Quantity | Units |
| --- | --- | --- |
| i, f | Initial and final, e.g., referring to *H* or *t.* | n/a |
| *H*, *W*, *L* | Height, width and length. Strictly, *H* is bedform amplitude. | m |
| *t* | Time; *t*_1_ and *t*_2_ are earlier and later times respectively | s |
| $t_{N}$ | Net time spent growing | s |
| $t_{\text{g}}$, $t_{\text{s}}$ | Time growing, shrinking | s |
| a, b, c | Constants | m, m, s |
| α, β | Parameters of the Gamma distribution – WT model [M10]; α_obs_, β_obs_ are values of metrics estimated from observed size-frequency data. | no units, s^-1^ |
| μ, σ | Parameters of the log-normal distribution – SI model [M7]; μ_obs_, σ_obs_ are values of metrics estimated from observed size-frequency data. | no units |
| λ | Rate parameter for Poisson processes. | s^-1^ |
| λ_obs_, φ_obs_ | Exponent and mode of size-frequency data, as approximated in Hillier et al. (2013). | m^-1^, m |
| *k* | Growth rate constant | ms^-1^ or s^-1^ |
| *n* | Number of bedform observations. | no units |
| $k_{\text{g}}, k_{\text{s}}$ | Growth rates of growth and shrinking, when differentiated; see text for relation to $k_{\text{av}}, k_{\text{net}}$. | s^-1^ |
| *n*_b_ | Number of growth episodes – WT model [M10]. | no units |
| *j* | Number of bedforms in a patch | no units |
| *p* | Probability of growth | no units |
| $\xi$ | Statistical drift – SI model [M7] |  |
| *v* | Ice velocity | ms^-1^ |
| τ | Basal shear stress | Nm^-2^ |
