# Supplementary material for: Exploring Explanations of Subglacial Bedform Sizes Using Statistical Models
Source: PLoS One. 2016 Jul 26;11(7):e0159489. doi: 10.1371/journal.pone.0159489 (PMC4961447; doi:10.1371/journal.pone.0159489)
Supplement: S1 File — Also includes a summary table of notation used in the manuscript. (ZIP) [file pone.0159489.s001.zip › S1 File/Table_of_Notation.pdf]

Table of Notation

Symbol	Quantity	Units
i, f	Initial and final, e.g., referring to H or t.	n/a
H, W, L	Height, width and length. Strictly, H is bedform amplitude.	m
t	Time; t_1 and t_2 are earlier and later times respectively	s
t_N	Net time spent growing	s
t_g, t_s	Time growing, shrinking	s
a, b, c	Constants	m, m, s
α, β	Parameters of the Gamma distribution – WT model [M10]; α_{obs}, β_{obs} are values of metrics estimated from observed size-frequency data.	no units, s^{-1}
μ, σ	Parameters of the log-normal distribution – SI model [M7]; μ_{obs}, σ_{obs} are values of metrics estimated from observed size-frequency data.	no units
λ	Rate parameter for Poisson processes.	s^{-1}
λ_{obs}, ϕ_{obs}	Exponent and mode of size-frequency data, as approximated in Hillier et al. (2013).	m^{-1}, m
k	Growth rate constant	ms^{-1} or s^{-1}
n	Number of bedform observations.	no units
k_g, k_s	Growth rates of growth and shrinking, when differentiated; see text for relation to k_{av}, k_{net}.	s^{-1}
n_b	Number of growth episodes – WT model [M10].	no units
j	Number of bedforms in a patch	no units
p	Probability of growth	no units
ξ	Statistical drift – SI model [M7]	
v	Ice velocity	ms^{-1}
τ	Basal shear stress	Nm^{-2}
